# Matrix metalloproteinase-7 is dispensable in a mouse model of sepsis-induced acute lung injury

**DOI:** 10.1371/journal.pone.0321349

**Published:** 2025-05-08

**Authors:** Brandon Baer, Jason Lin, Kaitlyn R. Schaaf, Lorraine B. Ware, Ciara M. Shaver, Julie A. Bastarache

**Affiliations:** 1 Division of Allergy, Pulmonary and Critical Care Medicine, Department of Medicine, Vanderbilt University Medical Center, Nashville, Tennessee, United States of America; 2 Department of Pathology, Microbiology and Immunology, Vanderbilt University Medical Center, Nashville, Tennessee, United States of America; 3 Department of Cell and Developmental Biology, Vanderbilt University Medical Center, Nashville, Tennessee, United States of America; Stanford University, UNITED STATES OF AMERICA

## Abstract

Acute respiratory distress syndrome (ARDS) is a life-threatening form of acute lung injury whose pathogenesis is characterized by excessive lung inflammation and alveolar-capillary barrier permeability. Matrix metalloproteinase 7 (MMP7) can regulate leukocyte recruitment and the production of pro-inflammatory cytokines, but whether it plays a role in acute lung injury (ALI) is an unanswered question. We hypothesized that global loss of MMP7 would attenuate sepsis-induced ALI and systemic inflammation. To test this, male and female MMP7 knockout (MMP7KO) mice and wild-type (WT) littermates were exposed to a two-hit model of ALI (sepsis+hyperoxia). Sepsis was induced through intraperitoneal injection of cecal slurry (CS; 1.6mg/g) or 5% dextrose (control) followed by exposure to hyperoxia (HO; FiO_2_=0.95) or room air (control, FiO_2_=0.21). At 24-hours post-CS+HO, we measured weight loss, illness severity, and body temperature. The mice were then sacrificed, and samples from the lungs, kidneys, spleen, blood, peritoneal wash, and bronchoalveolar lavage (BAL) fluid were collected for analysis. Bacterial burden was assessed in the peritoneum, lung, and spleen. Lung inflammation was assessed by BAL inflammatory cell recruitment and pro-inflammatory cytokine concentrations as well as lung tissue mRNA expression of pro-inflammatory cytokines. Alveolar-capillary barrier disruption was quantified by BAL total protein, BAL immunoglobulin M, and lung wet-to-dry weight ratios. Histologic evidence of lung injury was evaluated using a histological scoring system. Systemic inflammation was measured through plasma pro-inflammatory cytokines and peritoneal inflammatory cells. Kidney function, inflammation, and injury were assessed through plasma urea nitrogen concentrations, as well as tissue levels of pro-inflammatory cytokines, neutrophil gelatinase-associated lipocalin (NGAL), and kidney injury molecule 1 (KIM-1). Relative mRNA expression of MMP-7, MMP-9, and MMP-2 was also quantified in both lung and kidney tissue through qPCR. At 24-hours post-CS+HO all mice developed ALI. Septic mice also had increased systemic inflammation, kidney inflammation, kidney injury, and kidney dysfunction compared to controls. Loss of MMP7 did not affect markers of inflammation, organ injury, or organ dysfunction. Interestingly, septic male mice exhibited more severe illness, systemic and lung inflammation, lung injury, and lung expression of matrix metalloproteinases, while septic female mice exhibited more kidney inflammation, kidney injury, and kidney expression of matrix metalloproteinases. In conclusion, MMP7 is not essential for the development or resolution of sepsis-induced ALI in this model and likely plays a limited role in the condition.

## Introduction

Acute Respiratory Distress Syndrome (ARDS) is a syndrome, characterized clinically by low blood oxygen levels and acute respiratory failure, and pathophysiologically by acute inflammation and loss of alveolar-capillary barrier integrity [1–3]. It accounts for 10% of ICU admissions and has a mortality rate of 30–50% [1–3]. ARDS is a syndrome with no specific therapies other than supportive care, thus insights into ARDS pathogenesis are necessary to move towards targeted therapies. Excessive inflammation represents a key aspect of ARDS and is strongly associated with poor outcomes [4,5] but the mechanisms regulating lung inflammation in ARDS are not completely understood.

The most common cause of ARDS is sepsis, a dysregulated host response to infection whereby widespread systemic inflammation leads to life-threatening organ dysfunction including dysfunction of the lungs and kidneys [2]. In the lungs, sepsis causes severe lung inflammation, marked by increased pro-inflammatory cytokines in the bronchoalveolar lavage (BAL) fluid and immune cell recruitment [3,6]. This inflammation damages the alveolar-capillary barrier, causing protein-rich fluid to enter the alveoli and impair blood oxygenation [3,6]. Overwhelming inflammation from sepsis can also damage the kidneys [7–9], reducing their function and causing waste products such as urea nitrogen to build up in the blood [9,10]. More research is needed to better understand the mechanisms that regulate the inflammatory response in sepsis-induced ARDS.

Matrix metalloproteinase-7 (MMP7) is a zinc-dependent proteolytic enzyme mainly produced by epithelial cells the lungs, gut, and kidney [11]. It plays a role in several physiological processes including extracellular matrix remodeling, tissue repair, and cell migration [11]. In septic and ARDS patients, MMP7 levels are elevated and are linked to increased inflammation, poorer patient outcomes, and higher mortality [12]. Specifically, plasma MMP7 is elevated in septic patients, while BAL and lung tissue MMP7 are elevated in ARDS patients [12–17]. Although its role in ARDS is not fully understood, MMP7 regulates inflammation and mortality in cecal ligation and puncture, intraperitoneal lipopolysaccharide (LPS), and intratracheal bleomycin-induced models of systemic inflammation, lung inflammation, and acute lung injury (ALI) [16,18,19]. Mice without MMP7 are protected from lethal doses of lipopolysaccharide (LPS)-induced systemic inflammation, showing reduced intestinal permeability and lung inflammation [18]. MMP7KO mice are also protected against immune cell influx, alveolar-flooding, and lung fibrosis associated with bleomycin-induced ALI [19]. Together, these data suggest a potential role for MMP7 in the pathogenesis of sepsis-induced ARDS. Thus, we hypothesized that global loss MMP7 would attenuate sepsis-induced ALI and non-pulmonary organ dysfunction through reduced systemic and pulmonary inflammation.

## Materials & methods

### Animals

Vanderbilt University Medical Center (VUMC) Institutional Animal Care and Use Committee (Protocol Number: M1600006-02) reviewed and approved mouse studies. Male and female mice lacking the expression of MMP7 were originally purchased from Jackson Laboratory (Bar Harbor, ME; JAX stock #005111; B6.129-MMP7^tm1Lmm^/J) [20], and were back-crossed with C57/BL6 MMP7^+/+^ mice to initiate and re-derive a pathogen free colony of MMP7^+/+^ (wild-type littermates) and MMP7^-/-^ (MMP7KO) mice [21]. Genotypes were confirmed using standard PCR as recommended by Jackson Laboratory. All experimental procedures were performed in both male and female mice at 8- to 15-weeks of age. All mice were group housed in a research animal facility, exposed daily to a 12-hour light/dark cycle, and had ad libitum access to food and water.

### Cecal slurry

Cecal Slurry (CS) was prepared as previously described [22,23]. Briefly, 6-week-old female C57BL/6 donor mice from Jackson Laboratory (Bar Harbor, ME) were euthanized upon arrival at VUMC. Their cecal contents were collected and pooled, then resuspended in 5% dextrose and filtered through a 70-µm cell strainer. To reduce batch to batch variability, a single large batch of CS was prepared, aliquoted for storage at -80°C, and used for all experiments.

### Experimental procedure and sample processing for animal experiments

Polymicrobial abdominal sepsis was induced through intraperitoneal injection of CS at 1.6 mg/g body weight or control solution of 5% dextrose. This was followed by 24-hours of hyperoxia (HO; FiO2 = 0.95) or room air (FiO2 = 0.21; control). Our previous studies have demonstrated that this clinically relevant two-hit model results in moderate ALI [22,23]. Antibiotics (0.3ml IP; Imipenem and Cilastatin at 5 mg/ml) and fluids (0.7ml saline; SQ) were administered 12-hours after CS+HO treatment. Observers who were blinded to treatment group assessed sepsis illness severity every 4-hours using a validated semi-quantitative sepsis scoring system [22]. Mice were immediately euthanized if immobility or a sepsis severity score less than or equal to five was observed. Mice were euthanized by IP injection of sodium pentobarbital (Sigma-Aldrich, St. Louis, MO). Blood was collected from the right ventricle via cardiac puncture using a 23-gauge needle and collected in K2EDTA tubes (Becton, Dickinson and Company, Franklin Lakes, NJ). Plasma was separated by centrifugation at 1,000 g for 10 minutes at 4ºC. Peritoneal fluid was collected in 5 ml of sterile saline, centrifuged at 1,000 g for 10 minutes at 4ºC, and its supernatant was stored at -80°C.

Euthanized mice were then divided into two groups. In the first group, the left lung was weighed and then dried at 55°C for 72 hours to calculate wet-to-dry weight ratio. The right lung was used to collect BAL fluid in 0.6 mL of sterile saline, centrifuged at 1,000 g for 10 minutes at 4ºC, and supernatant was stored at -80°C. The right lung tissue was immediately flash frozen in liquid nitrogen, and stored at -80°C. In the second group, the right lung was fixed for histology, while the left lung, peritoneal fluid, and spleen were used to measure bacterial burden. For bacterial counts, organs were dipped in 100% ethanol to remove adherent bacteria before being homogenized, serially diluted in phosphate buffered saline, streaked onto LB agar plates, and incubated for 24 hours at 37°C. Colony forming units (CFU) of bacteria were counted and the data were natural log transformed prior to analysis. The lower limit of detection was 33 CFU/mL. For histology, the right lung was perfused with 10% heparin before being perfused with 4% paraformaldehyde (PFA) via the right ventricle. The right lung was inflated with 4% PFA through the trachea and then excised and stored in 4% PFA overnight. After embedding in paraffin and sectioning, lung sections were stained with hematoxylin and eosin.

### Experimental outcomes

#### Body weight and temperature.

Body weights were recorded prior to CS injection as well as 12- and 24-hour post-CS injection. Body temperatures were recorded prior to and every 4 hours post-CS injection using a Contec Infrared Thermometer model TP500 (Contec Co., Ltd. Osaka, Japan).

#### Lung inflammation and edema.

Cell counts were performed on fresh BAL using a light microscope and hemocytometer. Cell differentials were performed on cytospins of BAL using a light-based microscope and slides stained with Shandon™ Kwik-Diff™ Stains (Epredia, Portsmouth, NH). The concentration of BAL interleukin-1β (IL-1β), interleukin-6 (IL-6), interleukin-10 (IL-10), interleukin-12p70 (IL-12p70), tumor necrosis factor α (TNF-α), C-X-C motif chemokine ligand 1 (CXCL-1), and interferon-γ (IFN-γ) were measured with the V-PLEX Proinflammatory Panel 1 (Meso Scale Discovery, Rockville, MD) [24]. BAL total protein was measured by Pierce bicinchoninic acid protein assay (ThermoFisher Scientific, Rockford, IL). BAL immunoglobulin M (IgM) was quantified using an IgM Mouse Uncoated ELISA Kit (Invitrogen, Waltham, MA). Frozen lung tissue was homogenized to measure mRNA expression of MMP2, MMP7, and MMP9, as well as pro-inflammatory cytokines IL-6, IL-1β, IFN-γ, and CXCL-1. Briefly, mRNA extraction was performed using a Qiagen RNeasy Plus Mini Kit (Hilden, Germany), with cDNA generated through an iScript cDNA Synthesis Kit (Bio-Rad, Hercules, CA). MMP7 (12001950) expression was measured by qPCR and normalized to GAPDH (10031226) expression using Bio-Rad PrimePCR probes (Hercules, CA). MMP2 (Mm00439498_m1), MMP9 (Mm0042991_m1), IL-6 (Mm00446190_m1), IL-1β(Mm00434228_m1), IFN-γ (Mm01168134_m1), and CXCL-1(Mm04207460_m1) expression were also normalized to GAPDH (Mm99999915_g1) expression but using Taqman PrimePCR probes (ThermoFisher Scientific, Rockford, IL). A Ct value of 40 was used for samples with a non-detectable expression.

#### Histological lung injury.

Images of the two lobes of the right lung were captured with no overlap at 20x magnification (10 images total). Each image was evaluated in a blinded manner and assigned a score from 1 to 5 for four parameters: 1) inflammation, 2) septal thickening, 3) edema, and 4) red blood cells (RBC) in the alveolar space [22,23,25–28]. The scores for each parameter across the 10 images were then averaged to calculate an overall lung injury score.

#### Kidney injury and systemic inflammation.

Plasma was analyzed to determine blood urea nitrogen concentrations using a Urea Nitrogen Colorimetric Detection Kit from Invitrogen (Waltham, MA). Frozen kidney tissue was homogenized to measure mRNA and protein expression of injury markers neutrophil gelatinase-associated lipocalin (NGAL) and kidney injury marker-1 (KIM-1) as well as mRNA expression of MMP2, MMP7, MMP9, and pro-inflammatory cytokines IL-6, IL-1β, and CXCL-1 as described above. Briefly, mRNA extraction was performed using a Qiagen RNeasy Plus Mini Kit (Hilden, Germany), with cDNA generated through an iScript cDNA Synthesis Kit (Bio-Rad, Hercules, CA). NGAL (Lcn2; 10031226) and KIM-1 (Havcr1; 10031229) expression were measured by qPCR and normalized to GAPDH (10031226) expression using Bio-Rad PrimePCR probes (Hercules, CA). Protein levels of NGAL (Goat AF1857) and KIM-1 (Goat AF1817) were measured by western blot of kidney homogenate prepared in RIPA buffer (Thermo Scientific, Waltham, MA) using antibodies from R&D Systems (Minneapolis, MN), and normalized to β-actin (Rabbit 4970) from Cell Signaling Technology (Danvers, MA). The concentration of plasma IL-1β, IL-6, IL-10, IL-12p70, TNF-α, CXCL-1, and IFN-γ were measured with the V-PLEX Proinflammatory Panel 1 (Meso Scale Discovery, Rockville, MD) [24]. Peritoneal inflammation was evaluated through cell counts on fresh peritoneal fluid using a light microscope and hemocytometer. Cell differentials were performed on cytospin peritoneal fluid after slides were stained with Shandon™ Kwik-Diff™ Stains (Epredia, Portsmouth, NH).

### Randomization and data analysis

Cohorts of mice (each a combination of 1–3 litters) were randomized 1:2 into control or CS+HO treatment groups. A total of 9 separate cohorts were used in this study, with experiments being performed over multiple independent days. There were no exclusions for this study resulting in 76 mice being used: 43 WT mice (21 male mice [6 control and 15 CS+HO] and 22 female mice [6 control and 16 CS+HO]) and 33 MMP7KO mice (17 males [6 control and 11 CS+HO] and 16 females [4 control and 12 CS+HO]). Statistical analysis was performed using GraphPad Prism version 9 (GraphPad Software, Inc., La Jolla, CA). For non-normally distributed data, comparison of two groups was made using Mann-Whitney U test, while comparison of more than two groups was made using Kruskal-Wallis test with a Dunn’s multiple comparisons test. For data collected over multiple timepoints, a repeated Two-way ANOVA with a Tukey’s multiple comparisons test was utilized. A p-value of less than 0.05 was considered statistically significant.

## Results

### MMP7KO does not affect sepsis-induced illness severity, hypothermia, weight loss, bacterial burden, or systemic inflammation

**Fig 1:**
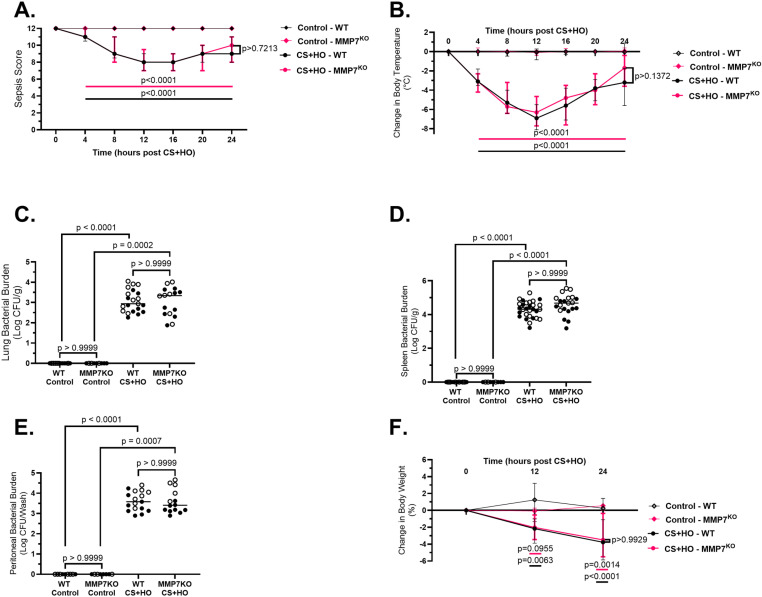
Severity of illness, hypothermia, bacterial burden, and weight loss over the course of a 24-hour septic insult [cecal slurry (CS; 1.6mg/g) + hyperoxia (HO; 95% O_2_)] in MMP7KO mice and their wild-type littermates. Septic mice (in both WT and MMP7KO) showed significantly greater illness severity (A) and loss of body temperature (B) at all timepoints after 4 hours post-CS+HO, as well as spleen bacterial burden (C), peritoneal bacterial burden (D), and lung bacterial burden (E) at 24-hours post CS+HO compared to control mice. WT septic mice also showed significantly greater weight loss compared to WT control mice at all 12-hours and 24-hours post CS+HO (F). MMP7KO septic mice had significantly greater weight loss at 24-hours post CS+HO and numerically greater weight loss compared to MMP7KO control mice. No differences were observed across the genotypes for septic mice any of the measured outcomes. N = 9-29. [Statistical analysis: Repeated Two-way ANOVA (A, B, F); Kruskal-Wallis test with a Dunn’s multiple comparisons test (C-E)]. Each point represents either the combined median of male and female animals (A, B, F) or an individual male (solid circle) or female (open circle) animal (D-F). Error bars indicate interquartile range, while horizontal lines represent statistical comparisons between septic WT (black) or MMP7KO (pink) and their respective controls (A, B, F). while horizontal line indicates combined median of male and female animals (C-E). Control = 5% dextrose + room air at 21% O_2_.

**Fig 2:**
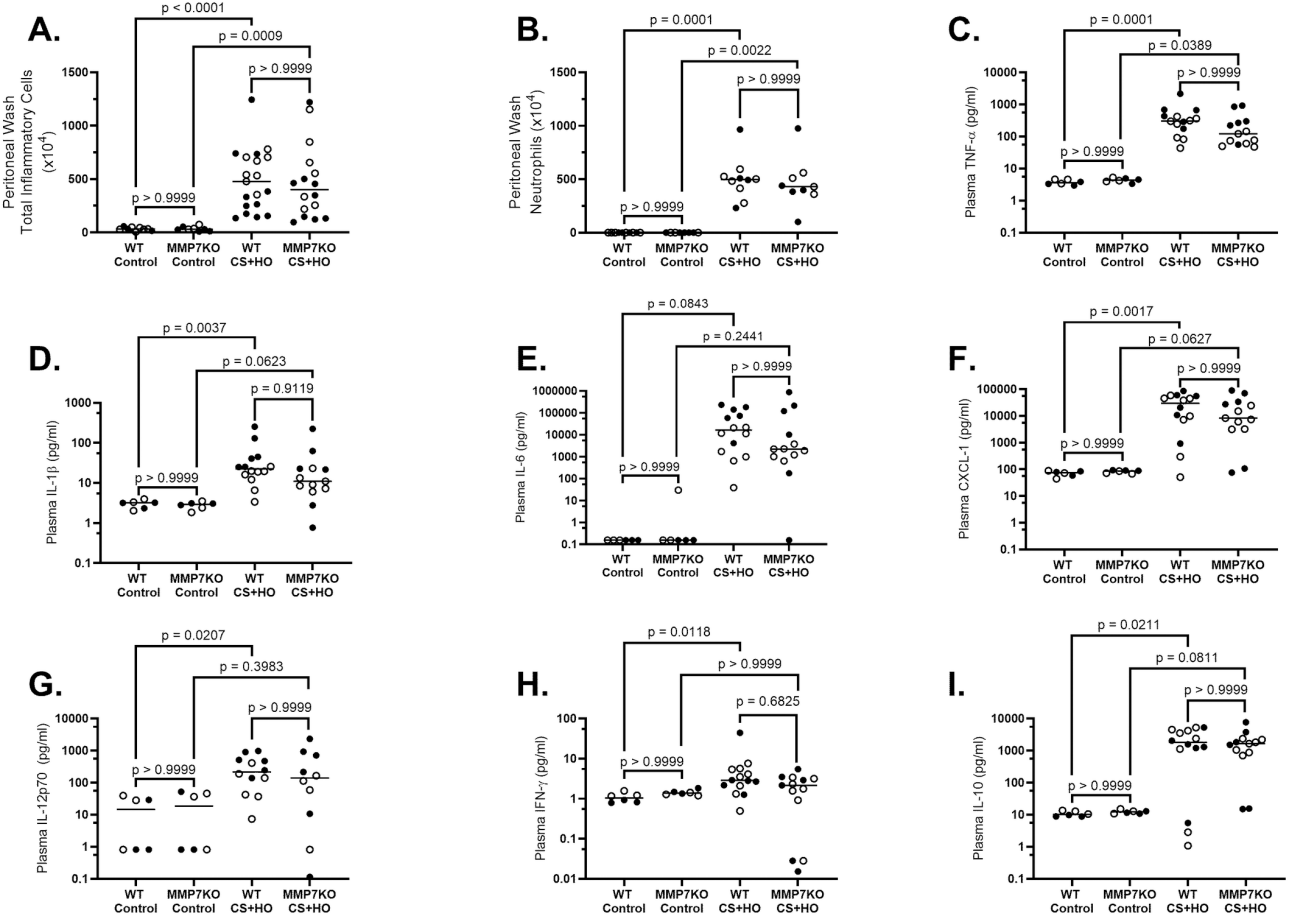
Systemic Inflammation associated with a 24-hour septic insult [cecal slurry (CS; 1.6mg/g) + hyperoxia (HO; 95% O_2_)] in MMP7KO mice and their wild-type littermates. Septic WT mice had significantly higher peritoneal wash inflammatory cell counts (A), peritoneal wash neutrophil counts (B), and plasma concentrations of concentrations of TNF-α (C), IL-1β (D), IL-6 (E), CXCL-1 (F), IL-12p70 (G), IFN-γ (H), and IL-10 (I) compared to their control mice. Similar significant increases were observed for septic MMP7KO mice compared to their controls, except for plasma IL-12p70 (G), IFN-γ(H). In septic MMP7KO mice plasma IL-10 (I; p =0.0811) trended towards an increased concentration compared to MMP7KO control mice. No differences were observed between MMP7KO and WT mice in either treatment group. N = 6-19. [Statistical analysis: Kruskal-Wallis test with a Dunn’s multiple comparisons test]. Each point represents an individual male (solid circle) or female (open circle) animal. Horizontal line indicates combined median of male and female animals. Control = 5% dextrose + room air at 21% O_2_. TNF-α = tumor necrosis factor-α, IL-12p70 = interleukin-12p70, IL-6 = interleukin-6, IL-1β = interleukin-1β, and CXCL-1 = C-X-C motif chemokine ligand 1, IFN-γ = interferon gamma, IL-10 = interleukin-10.

Genetic deletion of MMP7, as confirmed by non-detectable lung or kidney MMP7 mRNA expression across control and septic treatment groups (S1 Fig), did not alter any markers of physiological dysfunction for sepsis-induced ALI. Despite CS+HO inducing significant increases for all markers of physiological dysfunction in both genotypes compared to controls, no differences were observed between MMP7KO and WT mice for illness severity, body temperature, weight loss, or bacterial burden, as well as in the spleen, peritoneal fluid, or lung tissue in either control or CS+HO groups in females (S2 Fig), males (S3 Fig), or the combination of both sexes (Fig 1). Septic mice (both WT and MMP7KO) also showed significant increases across multiple markers of systemic inflammation, however MMP7KO mice had similar levels of systemic inflammation compared to their WT littermates as measured by peritoneal inflammatory cell counts, peritoneal neutrophils, and plasma pro-inflammatory cytokine concentrations in females (S3 Fig), males (S4 Fig), or the combination of both sexes (Fig 2). For sex differences across these outcomes, septic WT males had a greater drop in body temperature, increased illness severity, and higher plasma concentrations of pro-inflammatory cytokines (TNF- α, IL-6, IL-1β, and IL-12p70) compared to septic female WT mice (Table 1). However, female WT mice showed greater sepsis-induced weight loss and a higher bacterial burden in the lung and peritoneal wash compared to septic male mice. Similar sex differences were observed in septic MMP7KO mice (Table 2).

**Table 1:** Sex differences for septic WT mice at 24-hour timepoint.

Outcome [n-value/group]	Male-CS+HO Median (IQR)	Female-CS+HO Median (IQR)	P-value
**Illness Severity [n = 9–17]**			
Change in Body Weight (%)	-1.27 (-4.04, 0.11)	-5.54 (-6.27, -3.28)	0.0035
Sepsis Score	9 (7.5, 9)	11 (10, 11)	<0.0001
Change in Body Temperature (°C)	-3.4 (-7.6, -1.8)	-2.3 (-3.3, -1.5)	0.0835
**Bacterial Burden [n = 8–16]**			
Spleen (Log CFU/g)	4.3 (3.84, 4.32)	4.44 (3.93, 4.73)	0.4753
Peritoneal Wash (Log CFU/wash)	3.23 (3.04, 3.74)	3.96 (3.55, 4.15)	0.0464
Lung Bacterial Count (Log CFU/g)	2.78 (2.51, 3.28)	3.19 (2.81, 3.89)	0.0845
**Lung Inflammation [n = 7–11]**			
BAL Total Inflammatory Cell Count (x10^4^ cells)	6.25 (4.81, 8.38)	7.25 (3.5, 10)	0.8248
BAL Neutrophils (x10^4^ cells)	0 (0, 0)	0 (0, 0)	>0.9999
BAL TNF-α (pg/ml)	5.13 (3.23, 44.74)	6.5 (2.58, 761.4)	0.8125
BAL IL-6 (pg/ml)	279.1 (97.54, 1295)	271.4 (40.05, 3952)	0.8125
BAL IL-1β (pg/ml)	0.82 (0.52, 3.97)	6.38 (1.36, 39.43)	0.0702
BAL CXCL-1 (pg/ml)	193.2 (49.44, 513.5)	145.8 (57.1, 438.4)	0.8125
BAL IFN-γ(pg/ml)	0.12 (0.11, 0.64)	0.1 (0.03, 21.47)	0.6678
BAL IL-12p70 (pg/ml)	7.18 (5.57, 16.58)	45.74 (0.82, 144)	0.5335
BAL IL-10 (pg/ml)	4.15 (1.32, 8.19)	4.77 (2.93, 14.33)	0.8868
Lung IL-6 mRNA (2^-ΔΔCt^)	5.87 (3.95, 9.06)	4.46 (1.11, 8.16)	0.5697
Lung IL-1β mRNA (2^-ΔΔCt^)	30.94 (26.36, 45.98)	17.93 (13.89, 35.18)	0.1939
Lung CXCL-1 mRNA (2^-ΔΔCt^)	521075 (170488, 656726)	135760 (31733, 365483)	0.1939
Lung IFN-γmRNA (2^-ΔΔCt^)	4.76 (2.9, 5.2)	2.08 (1.78, 3.44)	0.1333
Lung MMP2(mRNA (2^-ΔΔCt^)	9.96 (8.54, 20.51)	5.82 (4.48, 9.1)	0.0848
Lung MMP9(mRNA (2^-ΔΔCt^)	5.87 (3.45, 9.09)	12.33 (11.69, 15.15)	0.0040
Lung MMP7(mRNA (2^-ΔΔCt^)	0.98 (0.56, 1.66)	3.51 (1.8, 7.58)	0.0485
**Alveolar–Capillary Barrier Integrity [n = 7–11]**			
Lung Wet-to-Dry Weight Ratio	5 (4.64, 5.55)	5 (4.4, 5.5)	0.8757
BAL Protein (μg/ml)	217.1 (132.3, 276.9)	251.1 (231.6, 326)	0.0693
BAL IgM (μg/ml)	23.85 (17.46, 40.95)	43.58 (27.12, 89.77)	0.1220
**Histological Lung Injury [n = 5]**			
Total Lung Injury Score	2.7 (2.2, 3.15)	3 (2.65, 3.4)	0.3413
Inflammation	1.2 (0.95, 1.35)	1.1 (1, 1.3)	0.7222
Septal Thickening	0.8 (0.75, 1.05)	1.3 (0.9, 1.6)	0.1429
Edema	0.2 (0.1, 0.55)	0.3 (0.15, 0.35)	0.8810
RBCs in Airspace	0.3 (0.25, 0.45)	0.4 (0.15, 0.55)	0.7063
**Systemic Inflammation [n=6–13]**			
Peritoneal Total Inflammatory Cell Count (x10^4^)	249 (149.6, 559.4)	536.3 (291.4, 705)	0.3553
Peritoneal Neutrophils (x10^4^)	361.7 (167.3, 622.4)	449.3 (147.5, 519.4)	0.9497
Plasma TNF-α (pg/ml)	550 (259.3, 1054)	272.3 (84.59, 350.3)	0.0593
Plasma IL-6 (pg/ml)	107100 (45026, 195584)	6527 (736.4, 18462)	0.0080
Plasma IL-1β (pg/ml)	42.79 (25.15, 160.8)	17.6 (7.987, 19.76)	0.0027
Plasma CXCL-1 (pg/ml)	38001 (8291, 66354)	24048 (2012, 45307)	0.2824
Plasma IFN-γ(pg/ml)	2.746 (1.949, 13.27)	3.834 (1525, 5.616)	0.7546
Plasma IL-12p70 (pg/ml)	493.2 (218.9, 921.2)	91.36 (29.61, 245.0)	0.0260
Plasma IL-10 (pg/ml)	1439 (905, 2850)	2964 (283.3, 4440)	0.8518
**Kidney Damage, Inflammation, and Dysfunction [n=3–11]**			
Kidney MMP7 mRNA (2^-ΔΔCt^)	14.81 (6.9, 150.9)	8535 (3009, 14859)	0.0012
Kidney MMP2 mRNA (2^-ΔΔCt^)	0.56 (0.44, 0.84)	1.06 (0.79, 1.64)	0.0203
Kidney MMP9mRNA (2^-ΔΔCt^)	3.98 (2.98, 6.59)	6.48 (5.72, 10.01)	0.0992
Kidney NGAL mRNA (2^-ΔΔCt^)	546.6 (247.9, 848)	1412 (565, 1796)	0.0121
Kidney KIM-1 mRNA (2^-ΔΔCt^)	27.71 (10.89, 81.44)	381.6 (199.6, 458.7)	0.0012
Kidney NGAL Protein (Relative Expression)	41421 (37353, 43942)	73202 (54544, 93373)	0.1000
Kidney KIM-1 Protein (Relative Expression)	200.8 (147.7, 640.0)	1101 (461, 2451)	0.2000
Kidney IL-6 mRNA (2^-ΔΔCt^)	2.16 (1.9, 5.13)	4.05 (2.77, 4.53)	0.4173
Kidney IL-1β mRNA (2^-ΔΔCt^)	3.56 (2.1, 4.73)	3.29 (2.05, 4.47)	0.8968
Kidney CXCL-1 mRNA (2^-ΔΔCt^)	196.2 (46.92, 328.6)	171.4 (46.21, 440.4)	>0.9999
Plasma BUN (mg/dL)	91.82 (63.03, 100.1)	82.96 (43.24, 103.3)	0.7959

Definition of Abbreviations: IQR = interquartile range, CFU = colony forming units, g = gram, BAL = bronchoalveolar lavage, TNF-α = tumor necrosis factor-α, IL-6 = interleukin-6, IL-1β = interleukin-1β, and CXCL-1 = C-X-C motif chemokine ligand 1, IFN-γ = interferon gamma, IL-10 = interleukin-10, IgM = Immunoglobulin M, RBCs = red blood cells, MMP7 = matrix metalloproteinase-7, MMP2 = matrix metalloproteinase-2, MMP9 = matrix metalloproteinase-2, NGAL = neutrophil gelatinase-associated lipocalin, KIM-1 = kidney injury marker-1, BUN = blood urea nitrogen.

**Table 2:** Sex differences for septic MMP7KO mice.

Outcome [n-value/group]	Male-CS+HO Median (IQR)	Female-CS+HO Median (IQR)	P-value
**Illness Severity [n = 8–11]**			
Change in Body Weight (%)	-1.71 (-4.08, -0.13)	-4.75 (-7.6, -3.5)	0.0295
Sepsis Score	9 (8, 10)	11 (9.25, 11.25)	0.0764
Change in Body Temperature (°C)	-4.2 (-4.2, -1.2)	-1.35 (-2.88, -0.3)	0.3227
**Bacterial Burden [n = 5–11]**			
Spleen (Log CFU/g)	4.34 (3.7, 4.8)	4.97 (4.65, 5.4)	0.0048
Peritoneal Wash (Log CFU/wash)	3.19 (3.07, 3.41)	4.42 (3.8, 4.58)	0.0020
Lung Bacterial Count (Log CFU/g)	2.75 (2.37, 3.58)	3.34 (2.46, 3.81)	0.7430
**Lung Inflammation [n = 7–9]**			
BAL Total Inflammatory Cell Count (x10^4^ cells)	5.25 (4.81, 6.94)	5.75 (1.5, 6.75)	0.8398
BAL Neutrophils (x10^4^ cells)	0 (0, 0)	0 (0, 0)	>0.9999
BAL TNF-α (pg/ml)	9.03 (3.56, 171.3)	1.42 (0.85, 5.07)	0.1014
BAL IL-6 (pg/ml)	453 (131.5, 2238)	9.31 (0.16, 27.88)	0.0338
BAL IL-1β (pg/ml)	1.38 (0.57, 6.94)	0.73 (0.58, 2.364)	0.7551
BAL CXCL-1 (pg/ml)	191.3 (101.8, 920.7)	26.46 (7.79, 48.89)	0.0350
BAL IFN-γ(pg/ml)	0.13 (0.11, 0.6)	0.03 (0.03, 0.09)	0.0297
BAL IL-12p70 (pg/ml)	12.71 (10.94, 41.77)	0.82 (0.82, 730.7)	0.2429
BAL IL-10 (pg/ml)	4.33 (3.72, 29.4)	3.6 (0.12, 7.44)	0.5058
Lung IL-6 mRNA (2^-ΔΔCt^)	4.939 (3.82, 6.19)	2.84 (0.62, 5.26)	0.3524
Lung IL-1β mRNA (2^-ΔΔCt^)	19.36 (14.27, 25.27)	12.53 (6.72, 25.46)	0.4762
Lung CXCL-1 mRNA (2^-ΔΔCt^)	173764 (98601, 181195)	36082 (3672, 86431)	0.0317
Lung IFN-γ(mRNA (2^-ΔΔCt^)	3.28 (2.54, 4.81)	2.5 (0.71, 8.63)	0.4762
Lung MMP2(mRNA (2^-ΔΔCt^)	9.99 (4.37, 12.75)	5.86 (4.84, 7.86)	0.6095
Lung MMP9(mRNA (2^-ΔΔCt^)	7.94 (5.95, 10.53)	11.07 (8.38, 22.97)	0.1714
Lung MMP7(mRNA (2^-ΔΔCt^)	0.37 (0.19, 0.71)	0.35 (0.17, 0.52)	0.7619
**Alveolar–Capillary Barrier Integrity [n = 4–10]**			
Lung Wet-to-Dry Weight Ratio	5 (4.64, 5.55)	4.9 (3.87, 5.54)	0.5143
BAL Protein (μg/ml)	145.2 (131.3, 251.4)	216 (167.8, 249.7)	0.2105
BAL IgM (μg/ml)	45.47 (19.89, 58.36)	53.26 (39.53, 64.21)	0.4140
**Histological Lung Injury [n = 2–3]**			
Total Lung Injury Score	2.85 (2.5, 3.2)	2.6 (2.2, 3.7)	>0.9999
Inflammation	1.05 (0.9, 1.2)	1.1 (1.1, 1.2)	>0.9999
Septal Thickening	0.8 (0.6, 1)	0.9 (0.6, 1.6)	>0.9999
Edema	0.35 (0.3, 0.4)	0.4 (0.3, 0.5)	0.8000
RBCs in Alveolar Space	0.65 (0.3, 1)	0.1 (0.1, 0.6)	0.3000
**Systemic Inflammation [n=4–9]**			
Peritoneal Total Inflammatory Cell Count (x10^4^)	347.5 (126.9, 481.9)	553.8 (255, 847.5)	0.2105
Peritoneal Neutrophils (x10^4^)	144 (384.6, 439.8)	395.7 (85.78, 523.2)	>0.9999
Plasma TNF-α (pg/ml)	284.1 (181.8, 872.1)	73.9 (49.91, 121.8)	0.0221
Plasma IL-6 (pg/ml)	64971 (131.7, 381047)	1896 (1012, 2305)	0.3660
Plasma IL-1β (pg/ml)	22.15 (2.27, 103.4)	9.09 (7.28, 13.34)	0.5338
Plasma CXCL-1 (pg/ml)	30403 (99396, 74899)	7393 (3228, 15069)	0.3660
Plasma IFN-γ(pg/ml)	2.54 (0.03, 4.01)	1.77 (0.92, 3.18)	0.7617
Plasma IL-12p70 (pg/ml)	463.1 (8.11, 1284)	86.57 (15.46, 153.1)	0.3524
Plasma IL-10 (pg/ml)	1678 (15.5, 4757)	1659 (882.5, 2208)	0.9452
**Kidney Damage, Inflammation, and Dysfunction** **[n=4-11]**			
Kidney MMP7 mRNA (2^-ΔΔCt^)	0.55 (0.46, 0.88)	0.71 (0.28, 0.81)	0.9551
Kidney MMP2 mRNA (2^-ΔΔCt^)	0.35 (0.11, 0.77)	0.79 (0.43, 2.62)	0.1014
Kidney MMP9(mRNA (2^-ΔΔCt^)	5.15 (4.75, 6.28)	6.55 (4.45, 8.06)	0.5556
Kidney NGAL mRNA (2^-ΔΔCt^)	401.6 (125.7, 805.8)	565.2 (351.1, 1004)	0.2523
Kidney KIM-1 mRNA (2^-ΔΔCt^)	28.66 (11.89, 147.4)	97.24 (5.61, 336.6)	0.6806
Kidney NGAL Protein (Relative Expression)	36100 (23400, 48160)	58500 (26937, 70862)	0.4000
Kidney KIM-1 Protein (Relative Expression)	436 (89.79, 455.1)	1025 (668.5, 1597)	0.1000
Kidney IL-6 mRNA (2^-ΔΔCt^)	5.39 (3.98, 7.25)	3.99 (2.42, 5.73)	0.3524
Kidney IL-1β mRNA (2^-ΔΔCt^)	5.05 (4.19, 6.28)	2.07 (0.57, 4.51)	0.0401
Kidney CXCL-1 mRNA (2^-ΔΔCt^)	127.5 (77.84, 403)	16.53 (2.73, 183.7)	0.1419
Plasma BUN (mg/dL)	88.16 (67.66, 99.31)	74.16 (47.96, 91.2)	0.1331

Definition of Abbreviations: IQR = interquartile range, CFU = colony forming units, g = gram, BAL = bronchoalveolar lavage, TNF-α = tumor necrosis factor-α, IL-6 = interleukin-6, IL-1β = interleukin-1β, and CXCL-1 = C-X-C motif chemokine ligand 1, IFN-γ = interferon gamma, IL-10 = interleukin-10, IgM = Immunoglobulin M, RBCs = red blood cells, MMP7 = matrix metalloproteinase-7, MMP2 = matrix metalloproteinase-2, MMP9 = matrix metalloproteinase-2, NGAL = neutrophil gelatinase-associated lipocalin, KIM-1 = kidney injury marker-1, BUN = blood urea nitrogen.

### MMP7KO does not affect sepsis-induced ALI

**Fig 3:**
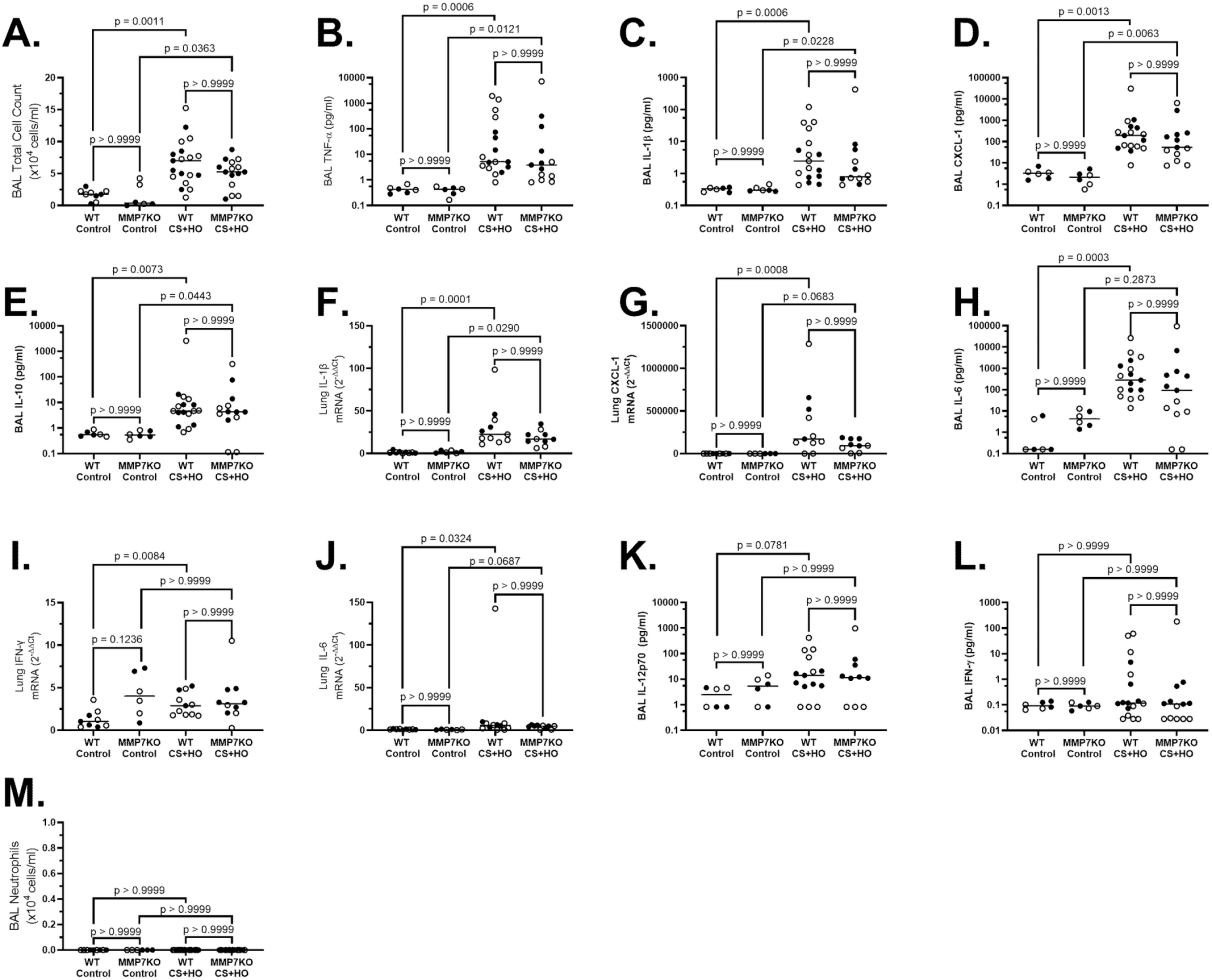
Lung inflammation induced by a septic insult [cecal slurry (CS; 1.6mg/g) + hyperoxia (HO; 95% O_2_)] in female and male MMP7KO mice compared to their wild-type littermates. Septic mice (in both WT and MMP7KO) had significantly higher BAL total cell counts (A), and BAL concentrations of TNF-α (B), IL-1β (C), and CXCL-1 (D), and IL-10 (E), as well as lung tissue IL-1β (F) and CXCL-1 (G) mRNA expression compared to their control mice. Septic WT mice also had significantly higher BAL concentrations of IL-6 (H), as well as lung tissue IFN-γ (I) and IL-6 mRNA (J) compared to WT controls, with BAL IL-12p70 (K) being numerical higher. Septic MMP7KO mice had numerically higher BAL IL-6 concentrations as well a lung tissue CXCL-1 and IL-6 mRNA compared to MMP7KO control mice, but did not differ with regards to lung tissue IFN-γmRNA expression or BAL concentrations of IL-12p70. Septic mice did not differ from control mice across either genotype for BAL IFN-γ (L) or BAL neutrophils (M). No differences between MMP7KO and WT genotypes were observed for any outcomes of lung inflammation in either treatment group. N = 6-19. [Statistical analysis: Kruskal-Wallis test with a Dunn’s multiple comparisons test]. Each point represents an individual male (solid circle) or female (open circle) animal. Horizontal line indicates combined median of male and female animals. Control = 5% dextrose + room air at 21% O_2_, BAL= bronchoalveolar lavage, TNF-α = tumor necrosis factor-α, IL-12p70 = interleukin-12p70, IL-6 = interleukin-6, IL-1β = interleukin-1β, and CXCL-1 = C-X-C motif chemokine ligand 1, IFN-γ = interferon gamma, IL-10 = interleukin-10.

**Fig 4:**
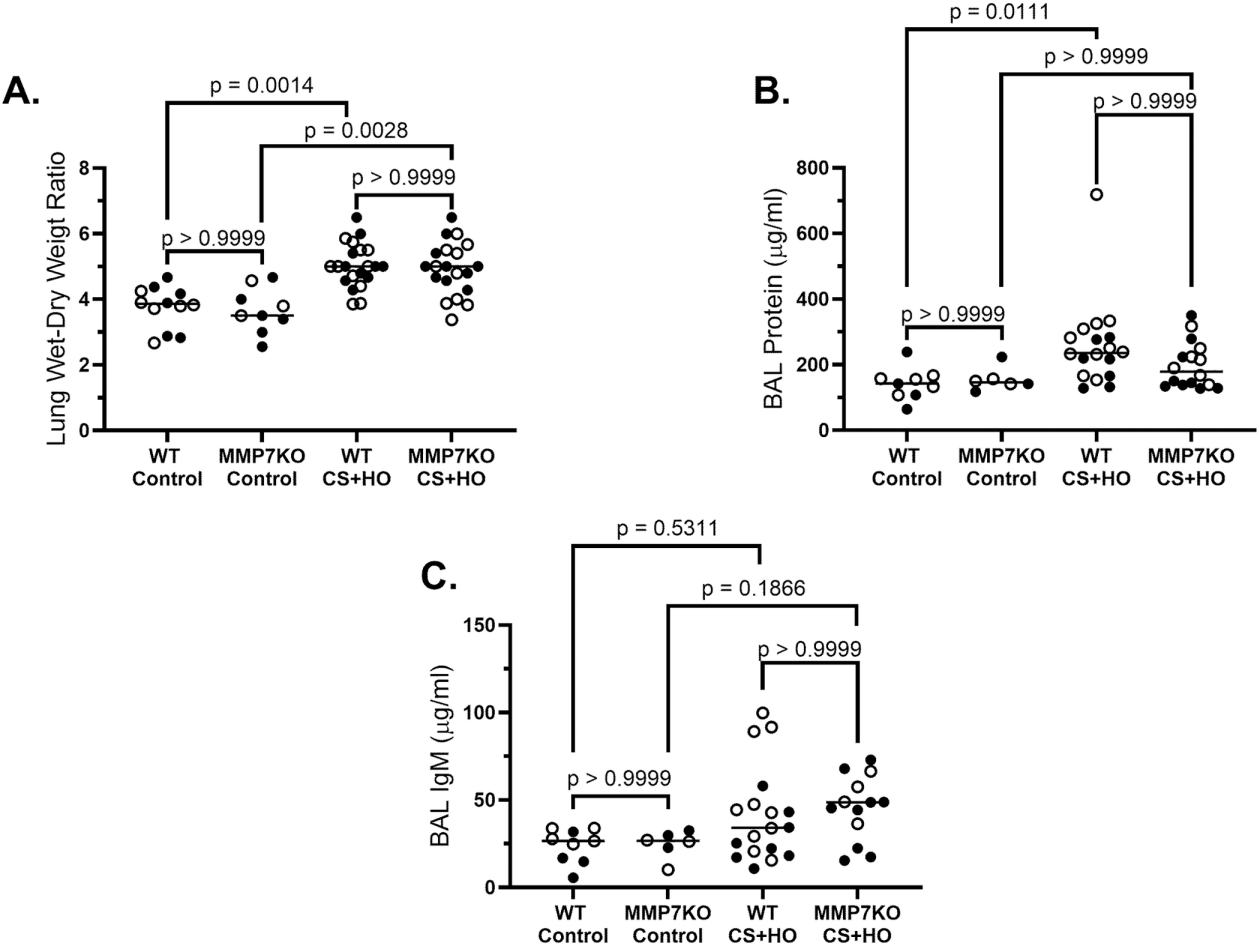
Alveolar-capillary barrier disruption induced by a septic insult [cecal slurry (CS; 1.6mg/g) + hyperoxia (HO; 95% O_2_)] in MMP7KO mice and their wild-type littermates. Compared to control mice, septic WT and MMP7KO mice showed significant increases in lung wet-to-dry weight ratios (A). Septic WT mice also showed significantly higher BAL protein compared to WT controls, while BAL protein in septic MMP7KO mice was not different compared to MMP7KO control mice (B). Septic MMP7KO mice also showed numerically higher BAL IgM compared to MMP7KO controls, while septic WT mice did not differ compared to control WT mice (C). No differences were observed between WT and MMP7KO mice in either treatment group. N = 6-21. [Statistical analysis: Kruskal-Wallis test with a Dunn’s multiple comparisons test]. Each point represents an individual male (solid circle) or female (open circle) animal. Horizontal line indicates combined median of male and female animals. Control = 5% dextrose + room air at 21% O_2_, BAL= bronchoalveolar lavage, IgM = immunoglobulin M.

**Fig 5:**
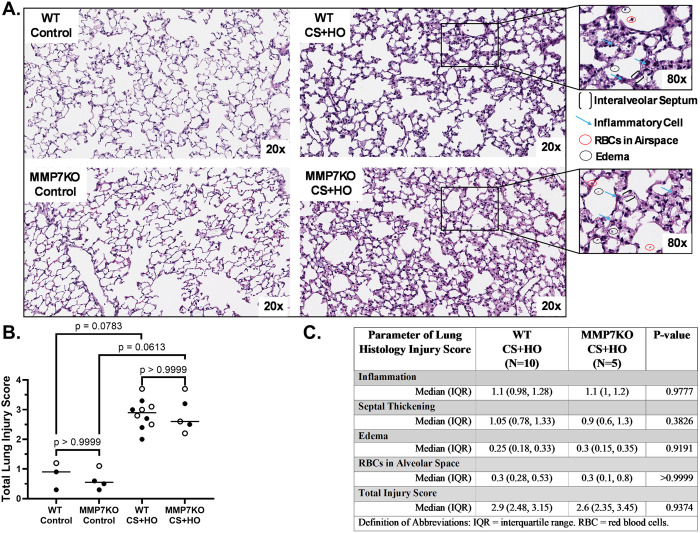
Histological Lung Injury induced by a septic insult [cecal slurry (CS; 1.6mg/g) + hyperoxia (HO; 95% O_2_)] in MMP7KO mice and their wild-type littermates. Representative whole lung sections in mice used for histology scoring (A). Lungs were stained with H&E and 20x images of the entire section scanned by the Vanderbilt University Medical Center Digital Histology Shared Resource using the Leica SCN400 Slide Scanner. Septic mice (WT and MMP7KO) had numerically higher total lung injury scores compared to their respective controls (B). No differences were observed between WT and MMP7KO mice in either treatment group. Individual parameters of lung histology injury score are presented as a table (C). N = 3-10. [Statistical analysis: (B) Kruskal-Wallis test with a Dunn’s multiple comparisons test; (C) Mann-Whitney U test]. Each point represents an individual male (solid circle) or female (open circle) animal. Horizontal line indicates combined median of male and female animals. Control = 5% dextrose + room air at 21% O_2_. RBC = red blood cell, IQR = interquartile range.

Septic mice had evidence of ALI compared to control; however, the MMP7KO genotype did not affect markers of lung inflammation, alveolar-capillary barrier permeability, or histological lung injury compared to WT littermates. In both control and septic mice, the total inflammatory cell counts and pro-inflammatory cytokine concentrations in BAL fluid, as well as pro-inflammatory cytokine mRNA in lung tissue were not different between MMP7KO and WT genotypes in females (S5 Fig), males (S6 Fig), or the combination of both sexes (Fig 3). In addition, no differences were observed between MMP7KO and WT mice across markers of alveolar-capillary barrier permeability including lung wet-to-dry weight ratio, BAL protein content, and BAL IgM concentration in females (S7 Fig), males (S8 Fig), or the combination of both sexes (Fig 4). Histological lung injury was also similar between septic MMP7KO and WT mice in females (S9 Fig), males (S10 Fig), or the combination of both sexes (Fig 5). No statistically significant sex differences were observed across markers of lung inflammation, alveolar-capillary barrier permeability or histological lung injury for septic WT mice (Table 1). However, although septic MMP7KO did not show sex differences across markers of alveolar-capillary barrier permeability or lung injury, septic male MMP7KO mice did have significantly higher concentrations of BAL IL-6, IFN-γ, and CXCL-1 compared to septic female MMP7KO mice (Table 2).

### MMP7KO does not affect sepsis-induced kidney injury

**Fig 6:**
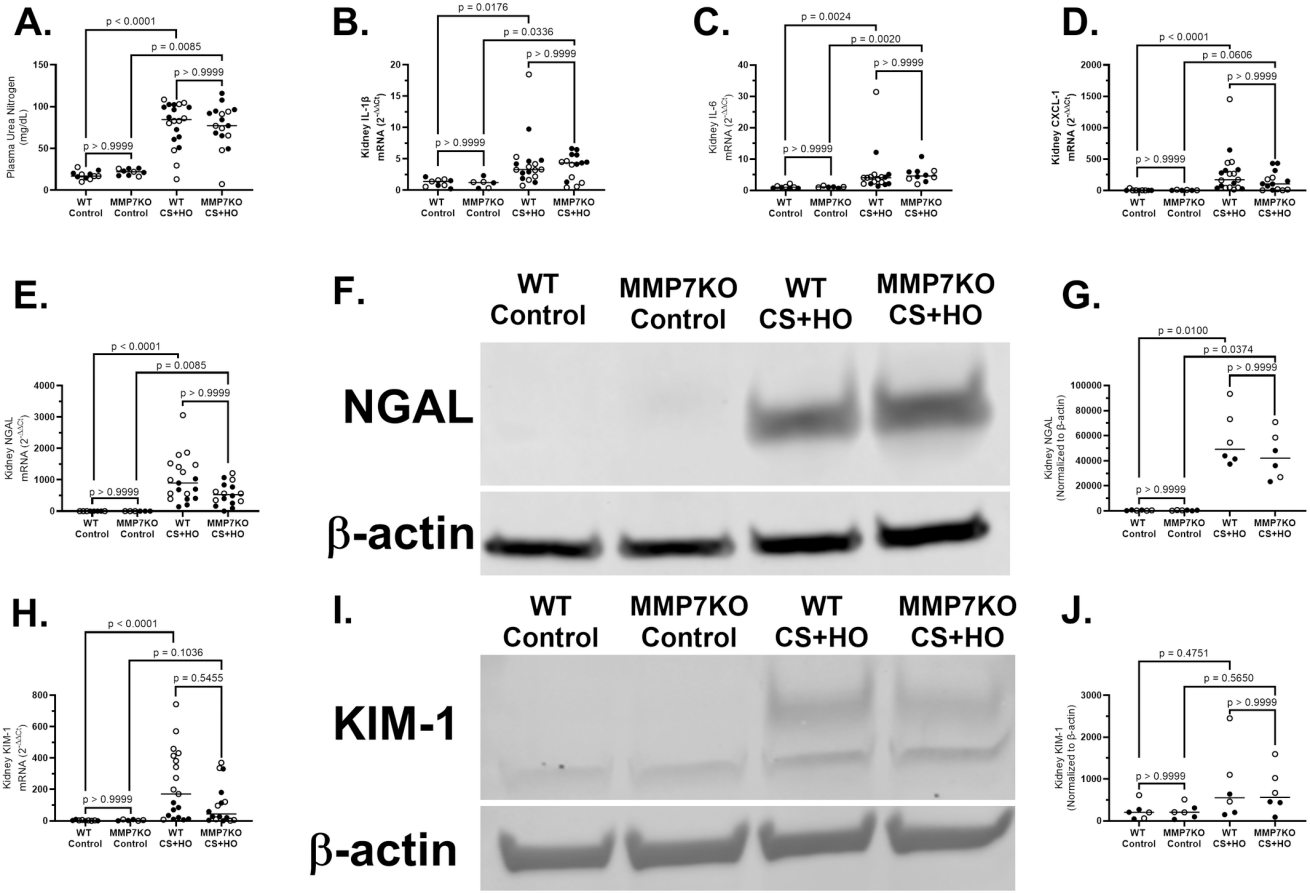
Kidney Dysfunction, Inflammation, and Injury induced by a septic insult [cecal slurry (CS; 1.6mg/g) + hyperoxia (HO; 95% O_2_)] in MMP7KO mice and their wild-type littermates. For both genotypes, septic mice showed significantly higher plasma urea nitrogen concentrations (A), kidney tissue mRNA expression of IL-1β (B), IL-6 (C), CXCL-1 (D), and NGAL (D), as well as kidney tissue NGAL protein (F) compared to control mice. Septic WT mice also had significantly higher kidney tissue mRNA expression of KIM-1, while septic MMP7KO had numerically higher KIM-1 mRNA (H). However, septic mice did not differ from control for kidney tissue KIM-1 protein in either genotype (G). No differences were observed between WT and MMP7KO mice in either treatment group. Representative western blots are displayed for NGAL (E) and KIM-1 (H) relative to β-actin. N = 6-20. [Statistical analysis: Kruskal-Wallis test with a Dunn’s multiple comparisons test]. Each point represents an individual male (solid circle) or female (open circle) animal. Horizontal line indicates combined median of male and female animals. Control = 5% dextrose + room air at 21% O_2_. IL-1β = interleukin-1β, IL-6 = interleukin-6, and CXCL-1 = C-X-C motif chemokine ligand 1, NGAL = neutrophil gelatinase-associated lipocalin, KIM-1 = kidney injury marker-1.

Markers of kidney dysfunction or injury did not differ between MMP7KO and WT mice with or without sepsis. Specifically, kidney dysfunction as measured by plasma blood urea nitrogen levels was similar between septic MMP7KO and WT mice in females (S11 Fig), males (S12 Fig), or the combination of both sexes (Fig 6). Septic MMP7KO and WT mice also had similar levels of kidney injury in females, males, or combination of both sexes as measured by NGAL and KIM-1 in kidney tissue at both the mRNA and protein level. No differences were observed between control MMP7KO and WT mice across any of the measured markers of kidney injury or dysfunction. For sex differences, septic WT females had significantly higher mRNA levels of NGAL and KIM-1 compared to septic WT male mice (Table 1), while septic male MMP7KO had significantly higher kidney IL-1β and numerically higher CXCL-1 mRNA expression compared to septic MMP7KO female mice (Table 2). However, no other sex differences were observed for markers of sepsis-induced kidney dysfunction, inflammation, or injury in either genotype.

### MMP7KO does not affect sepsis-induced MMP2 or MMP9 expression

**Fig 7:**
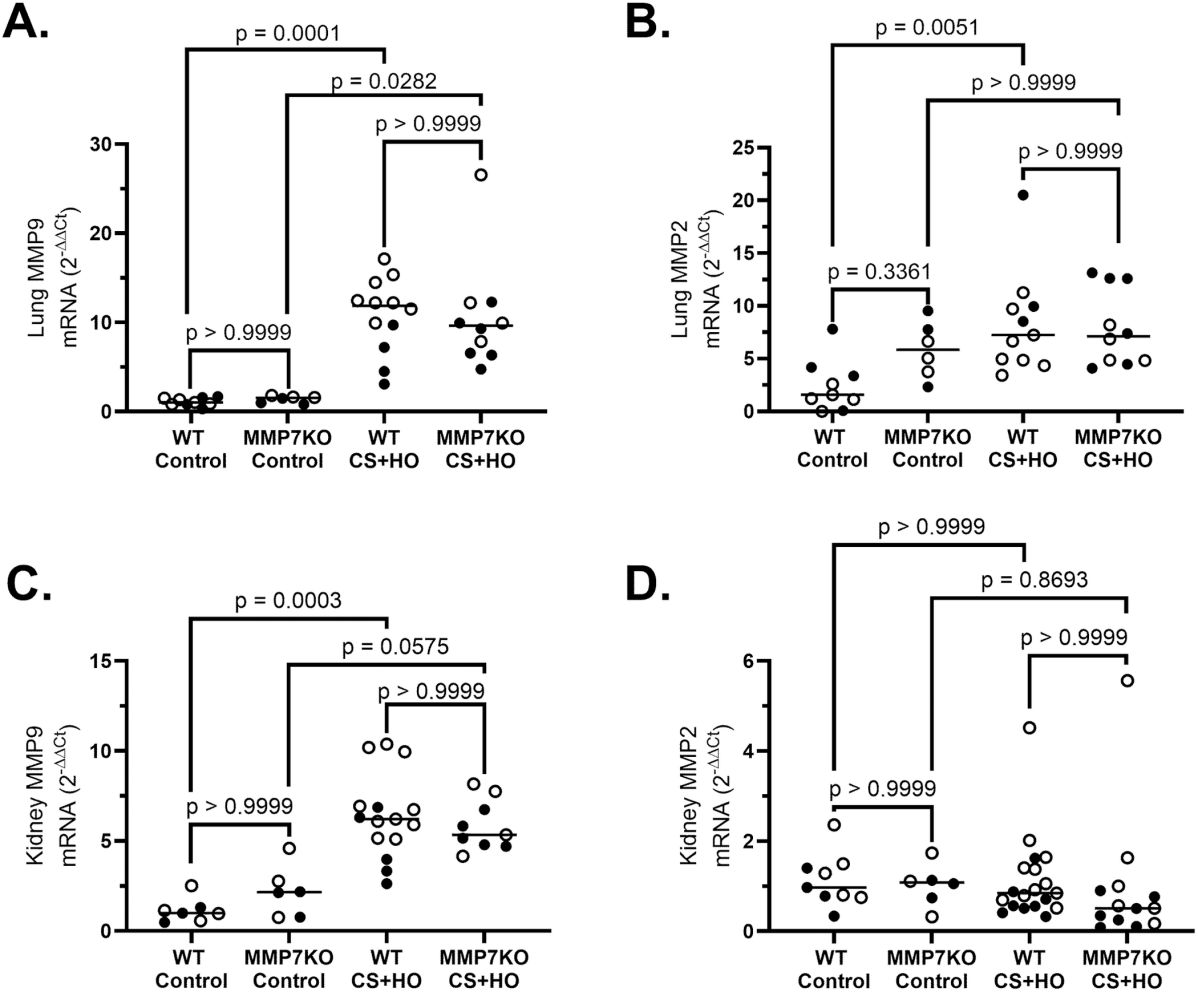
Lung and Kidney Matrix Metalloproteinase Expression induced by a septic insult [cecal slurry (CS; 1.6mg/g) + hyperoxia (HO; 95% O_2_)] in MMP7KO mice and their wild-type littermates. WT septic mice had significantly higher lung MMP9 (A) and MMP2 (C), as well as kidney MMP9 (E), but not kidney MMP2 (F) mRNA expression compared to WT control mice. Septic MMP7KO mice had significantly higher lung MMP9 and numerically higher kidney MMP9 compared to control MMP7KO mice. MMP7KO septic mice did not differ with regards to MMP9 or MMP2 in either organ. No differences were observed between genotypes for control mice. N = 6-21. [Statistical analysis: Kruskal-Wallis test with a Dunn’s multiple comparisons test]. Each point represents an individual male (solid circle) or female (open circle) animal. Horizontal line indicates combined median of male and female animals. Control = 5% dextrose + room air at 21% O_2_. MMP9 = matrix metalloproteinase-9, MMP2 = matrix metalloproteinase-2.

Despite CS+HO inducing significant increases in lung MMP2, lung MMP9 and kidney MMP9 expression for WT mice compared to control, no differences were observed between septic MMP7KO and septic WT mice across either MMP in females (S11 Fig), males (S12 Fig), or the combination of both sexes (Fig 7). Specifically, mRNA expression in lung and kidney tissue for MMP2 or MMP9 was similar between septic MMP7KO and WT mice in females, males, and the combination of both sexes. Regardless of sex, no significant differences were observed between control MMP7KO and WT mice across any of the MMPs measured in lung or kidney tissue. For sex differences, septic WT females had significantly higher lung mRNA levels of MMP7 and MMP9, as well as kidney mRNA expression of MMP7 and MMP2 compared to septic WT males (Table 1). Septic WT females also had numerically higher MMP2 mRNA in lung tissue and MMP9 mRNA in kidney tissue compared to septic WT male mice. Septic MMP7KO mice showed no sex differences across any of the MMPs measured in lung or kidney tissue (Table 2).

## Discussion

We investigated the role of MMP7 in ALI induced by polymicrobial abdominal sepsis and hyperoxia using global MMP7KO mice and their WT littermates. Following CS injection and exposure to HO, both genotypes showed weight loss, hypothermia, evidence of systemic illness, increased bacterial burden, systemic inflammation, lung inflammation, lung edema, lung expression of MMP9, and histologic lung injury compared to controls. Septic WT and MMP7KO mice also had increased kidney dysfunction, kidney inflammation, kidney expression of MMP9, and kidney injury compared to controls. While there were some measures that were numerically different in MMP7KO mice, none of these differences were statistically significant when compared to WT littermates and the biological relevance of these minor differences is not clear. Modest differences were also noted between male and female mice in both genotypes. Based on these data, we conclude that MMP7 has a limited role in the pathogenesis of murine sepsis-induced ALI and there may be sex differences in inflammatory responses to sepsis.

Many MMPs have been implicated in the pathogenesis of sepsis, kidney disease, and ARDS, promoting inflammation and disease severity [12,13,29–34]. We chose to focus on MMP7 in our study as it is an important regulator of kidney, systemic, and pulmonary inflammation in models of folic acid-induced kidney injury, LPS-induced systemic inflammation, and bleomycin-induced ALI [16,18,19,34]. In addition, its role in regulating these processes in a model of sepsis-induced ALI has not been studied. Although not significant, the modest numerical reductions in KIM-1 and NGAL in septic MMP7KO compared to septic WT mice raises the possibility that MMP7 could play a small role in sepsis associated kidney injury. Loss of MMP7 could potentially attenuate sepsis-induced kidney injury through decreased activation of NLRP3 and NLRP6 inflammasomes in the kidney [34]. However, loss of MMP7 in our model did not significantly alter any other measured outcomes including illness severity, systemic inflammation, lung inflammation, alveolar-capillary barrier permeability, lung injury, kidney dysfunction, or kidney inflammation compared to WT. One possible explanation for these findings is that other MMPs may compensate for the lack of MMP-7 in the current model of sepsis-induced ALI. Functional redundancy among MMPs, has been demonstrated previously [21,35,36], most notably with MMP2 and MMP9 [37,38]. For example, genetic deletion of MMP2 in mice results in a normal phenotype under physiological conditions, attributed to increased expression of MMP9 [38] Similarly, upregulation of MMP2 has been observed in MMP9KO mice in ALI models [37]. MMP2, MMP7, and MMP9 also share substrates such as heparan sulfate [39], supporting the potential for functional redundancy. Although our study did not observe differences in MMP9 or MMP2 mRNA expression in kidney or lung tissue between septic WT and MMP7KO mice, there were numerical differences between the genotypes for control mice. Specifically, the mRNA expression of both lung MMP2 (p=0.0593; Mann-Whitney U test) and kidney MMP9 (p=0.2343; Mann-Whitney U test) was more than 2-fold higher in control MMP7KO mice compared to control WT mice. Thus, although MMPs are not always functionally interchangeable, it is possible that one or more MMPs, likely MMP2 or MMP9, are compensating for the lack of MMP7 in the current model.

Another potential explanation for the discrepancies between the current study and those reporting protective effects for MMP7KO mice in lung injury is the differences in pathophysiological mechanisms driving direct (i.e., bleomycin) versus indirect (i.e., sepsis) lung injury [40]. Specifically, the lungs of MMP7KO mice are protected against direct bleomycin-induced lung injury [19,31,41]. MMP7 regulates processes such as pulmonary fibrosis, lung inflammation, and alveolar epithelial cell adhesion in direct lung injury models because it is constitutively expressed by alveolar epithelial cells and upregulated following direct injury to these cells [19,31,41,42]. In contrast, in systemically and other epithelial tissues, other MMPs, but not MMP7, regulate these processes in response to injury, with little change in MMP7 expression [31,43–45]. Essentially, MMP7 affects direct lung injury through alveolar epithelial cells, while other MMPs are responsible for the cell signaling outside the lung in indirect lung injury [31,44,45]. Our study suggests that the pulmonary protective effects of MMP7KO may be specific to direct lung injury.

Sex differences in response to CS+HO were observed across both genotypes in our two-hit model of sepsis-induced ALI. Septic male mice (both WT and MMP7KO) had higher levels of systemic inflammation markers (plasma TNF-α, IL-6, IL-1β, and IL-12p70) and lung inflammation markers (BAL IL-6, IFN-γ, and CXCL-1, as well as lung mRNA expression of IL-6, IL-1β, CXCL-1, and IFN-γ) compared to septic female mice. These finding align with the literature, which consistently reports higher levels of systemic inflammation markers in males compared to females in the context of sepsis [46]. Moreover, studies evaluating lung inflammation in both animals and humans have found that during the acute phase of respiratory disease, males have higher levels of pro-inflammatory cytokines and other markers of pulmonary inflammation compared to females [47]. In contrast, we found that septic WT females had higher lung mRNA expression of MMP7, kidney mRNA levels of MMP7 and MMP2, as well as kidney injury markers (KIM-1 and NGAL) compared to septic WT males. These differences were not observed in MMP7KO mice, suggesting that sex differences could be involved in some of the numerical differences between septic WT and MMP7KO mice, even if they did not reach statistical significance. Septic male mice (both WT and MMP7KO) also had increased illness severity including worse sepsis scores and greater drop in body temperatures across both genotypes in our study, while septic females showing greater weight loss and bacterial burden compared to septic males. Although female mice are generally considered more susceptible to CS-induced sepsis in terms of mortality, these findings are consistent with clinical observations. Male septic patients often experience worse outcomes, including a higher prevalence of complications, longer hospital stays, and higher mortality rates compared to females [46,48,49]. However, sex differences in outcomes across outcomes have not been consistently observed in ARDS patients, indicating that the influence of sex on sepsis and ARDS may differ [50,51].

The current study has some potential weaknesses. First, the lung injury induced by our two-hit model of sepsis-induced ALI is relatively mild. While it meets the relevant criteria for an experimental model of ALI [52], it does not result in robust recruitment of inflammatory cells, including neutrophils into the airspace [22,23]. MMP7 plays an important role in inflammation through direct cleavage of specific inflammatory mediators that generate chemotactic gradients for neutrophils [19,41]. Since neutrophils are important in ARDS pathophysiology [53], the current study leaves open the possibility that MMP7 may influence neutrophil-driven inflammation in ARDS through its proteolytic activity. Another limitation is that we did not evaluate intestinal permeability, bacterial translocation, or intestinal inflammation directly. Previous studies have suggested that the protective effects of MMP7KO in LPS-induced systemic inflammation are due to reduced intestinal permeability and bacterial translocation, leading to less systemic inflammation [18]. However, we did evaluate bacterial burden in the peritoneal space, lung, and spleen, finding no differences across genotypes. We also observed no differences in systemic inflammation between MMP7KO and WT septic mice, either in plasma or the peritoneal space. Additionally, our model utilized a live bacterial infection treated with antibiotics, rather than LPS. A previous study using a model of LPS-induced systemic inflammation found similar protective effects in WT mice treated with antibiotics as in MMP7KO mice [18]. Therefore, while MMP7 may be involved in bacterial translocation at the level of the intestines and LPS-induced inflammation, these effects seem to be redundant in the context of a live bacterial infection treated with antibiotics.

In summary, our findings address the role of MMP7 in the context of a clinically relevant model of sepsis-induced ALI. Our findings show that MMP7 does not play a significant role in ALI induced by polymicrobial abdominal sepsis, with MMP7KO mice showing similar sepsis-induced pathophysiology compared to WT mice. Notably, the absence of MMP7 does not significantly reduce sepsis-induced systemic, lung, or kidney inflammation, or affect the severity of lung or kidney injury severity of sepsis-induced kidney injury.

## Supporting information

S1 Fig Lung and Kidney MMP7 Expression induced by a septic insult [cecal slurry (CS; 1.6mg/g) + hyperoxia (HO; 95% O2)] in MMP7KO mice and their wild-type littermates. WT Septic mice had significantly higher lung (A) and kidney (B) MMP7 mRNA expression compared to WT control mice. No differences were observed between genotypes for control mice. N = 6–18. [Statistical analysis: Kruskal-Wallis test with a Dunn’s multiple comparisons test]. Each point represents an individual male (solid circle) or female (open circle) animal. Horizontal line indicates combined median of male and female animals. Control = 5% dextrose + room air at 21% O2. MMP7 = matrix metalloproteinase-7.
(TIF)

S2 Fig Severity of illness, hypothermia, bacterial burden, and weight loss over the course of a 24-hour septic insult [cecal slurry (CS; 1.6mg/g) + hyperoxia (HO; 95% O_2_)] in female MMP7KO mice and their wild-type littermates. Septic mice (in both WT and MMP7KO) showed significantly greater illness severity (A) and loss of body temperature (B) at all timepoints after 8 hours post-CS+HO, as well as spleen bacterial burden (C), peritoneal bacterial burden (D), and lung bacterial burden (E) at 24-hours post CS+HO compared to control mice. WT septic mice also showed significantly greater weight loss compared to WT control mice at all 12-hours and 24-hours post CS+HO (F). MMP7KO septic mice had numerically greater weight loss at 12-hours and 24-hours post CS+HO compared to MMP7KO control mice. No differences were observed across the genotypes for septic mice any of the measured outcomes. N = 3–13. [Statistical analysis: Repeated Two-way ANOVA (A, B, F); Kruskal-Wallis test with a Dunn’s multiple comparisons test (C-E)]. Each point represents either the median (A, B, F) or an individual animal with a horizontal line indicating median (D-F). Error bars indicate interquartile range, while horizontal lines represent statistical comparisons between septic WT (black) or MMP7KO (pink) and their respective controls (A, B, F). Control = 5% dextrose + room air at 21% O_2_.
(TIF)

S3 Fig Severity of illness, hypothermia, bacterial burden, and weight loss over the course of a 24-hour septic insult [cecal slurry (CS; 1.6mg/g) + hyperoxia (HO; 95% O_2_)] in male MMP7KO mice and their wild-type littermates. Septic mice (in both WT and MMP7KO) showed significantly greater illness severity (A) and loss of body temperature (B) at all timepoints after 8 hours post-CS+HO, as well as spleen bacterial burden (C), peritoneal bacterial burden (D), and lung bacterial burden (E) at 24-hours post CS+HO compared to control mice. WT septic mice also showed significantly greater weight loss at 24-hours post CS+HO (F) and numerically higher weight loss at 12 hours post CS+HO compared to control WT mice. MMP7KO septic mice had numerically greater weight loss at 12-hours, with significantly higher weight loss at 24-hours post CS+HO compared to MMP7KO control mice. No differences were observed between the genotypes for septic mice across any of the measured outcomes. N = 3–13. [Statistical analysis: Repeated Two-way ANOVA (A, B, F); Kruskal-Wallis test with a Dunn’s multiple comparisons test (C-E)]. Each point represents either the median (A, B, F) or an individual animal with a horizontal line indicating median (D-F). Error bars indicate interquartile range, while horizontal lines represent statistical comparisons between septic WT (black) or MMP7KO (pink) and their respective controls (A, B, F). Control = 5% dextrose + room air at 21% O_2_.
(TIF)

S4 Fig Systemic Inflammation associated with a 24-hour septic insult [cecal slurry (CS; 1.6mg/g) + hyperoxia (HO; 95% O_2_)] in female MMP7KO mice and their wild-type littermates. Septic WT mice had significantly higher peritoneal wash inflammatory cell counts (A), peritoneal wash neutrophil counts (B), and plasma concentrations of concentrations of TNF-α (C), and IL-6 (E) compared to their control mice. Female septic WT mice also had numerically higher plasma concentrations of IL-1β (D), CXCL-1 (F), IL-12p70 (G), IFN-γ (H), and IL-10 (I). Although female septic MMP7KO mice showed similar numerical increases across all pro-inflammatory cytokines, they were not significantly different compared to MMP7KO controls. No differences were observed between MMP7KO and WT mice in either treatment group. N = 3–8. [Statistical analysis: Kruskal-Wallis test with a Dunn’s multiple comparisons test]. Each point represents an individual animal. Horizontal line indicates median. Control = 5% dextrose + room air at 21% O_2_. TNF-α = tumor necrosis factor-α, IL-12p70 = interleukin-12p70, IL-6 = interleukin-6, IL-1β = interleukin-1β, CXCL-1 = C-X-C motif chemokine ligand 1, IFN-γ = interferon gamma, IL-10 = interleukin-10.
(TIF)

S5 Fig Systemic Inflammation associated with a 24-hour septic insult [cecal slurry (CS; 1.6mg/g) + hyperoxia (HO; 95% O_2_)] in male MMP7KO mice and their wild-type littermates. Septic WT mice had numerically higher peritoneal wash inflammatory cell counts (A), peritoneal wash neutrophil counts (B), and plasma concentrations of concentrations of TNF-α (C), and CXCL-1 (F) compared to their control mice. Male septic WT mice also had numerically higher plasma concentrations of IL-1β (D), IL-6 (E), IL-12p70 (G), IFN-γ (H), and IL-10 (I). Male septic MMP7KO mice showed significantly higher peritoneal inflammatory cell counts compared to control MMP7KO mice. Although male septic MMP7KO mice had similar numerical increases across all pro-inflammatory cytokines as WT, they were not significantly different compared to MMP7KO controls. No differences were observed between MMP7KO and WT mice in either treatment group. N = 3–11. [Statistical analysis: Kruskal-Wallis test with a Dunn’s multiple comparisons test]. Each point represents an individual animal. Horizontal line indicates median. Control = 5% dextrose + room air at 21% O_2_. TNF-α = tumor necrosis factor-α, IL-12p70 = interleukin-12p70, IL-6 = interleukin-6, IL-1β = interleukin-1β, CXCL-1 = C-X-C motif chemokine ligand 1, IFN-γ = interferon gamma, IL-10 = interleukin-10.
(TIF)

S6 Fig Lung inflammation induced by a septic insult [cecal slurry (CS; 1.6mg/g) + hyperoxia (HO; 95% O_2_)] in female MMP7KO mice compared to their wild-type littermates. Septic WT mice had significantly higher BAL total cell counts (A), BAL concentrations of TNF-α (B), IL-1β (C), CXCL-1 (D), and IL-6 (H), as well as lung tissue mRNA expression of IL-1b (F) and CXCL-1 (G) compared to their control mice. Septic WT also had numerically higher BAL IL-10 (E) and lung tissue mRNA expression of IFN-γ (I) compared to WT controls. Although septic MMP7KO mice showed similar numerical increases they were not significantly different compared to MMP7KO controls. Septic mice did not differ from control mice across either genotype for lung tissue mRNA expression of IFN-γ (I), IL-6 (J), BAL IL-12p70 (K), IFN-γ (L), or BAL neutrophils (M). No differences between MMP7KO and WT genotypes were observed for any outcomes of lung inflammation in either treatment group. N = 3–11. [Statistical analysis: Kruskal-Wallis test with a Dunn’s multiple comparisons test]. Each point represents an individual animal. Horizontal line indicates median. Control = 5% dextrose + room air at 21% O_2_, BAL= bronchoalveolar lavage, TNF-α = tumor necrosis factor-α, IL-12p70 = interleukin-12p70, IL-6 = interleukin-6, IL-1β = interleukin-1β, CXCL-1 = C-X-C motif chemokine ligand 1, IFN-γ = interferon gamma, IL-10 = interleukin-10.
(TIF)

S7 Fig Lung inflammation induced by a septic insult [cecal slurry (CS; 1.6mg/g) + hyperoxia (HO; 95% O_2_)] in male MMP7KO mice compared to their wild-type littermates. Male septic (both WT and MMP7KO) mice had numerically higher BAL total cell counts (A), BAL concentrations of TNF-α (B), IL-1β (C), CXCL-1 (D), IL-6 (H), and IL-12p70 (K) as well as lung tissue mRNA expression of CXCL-1 (G) and IL-6 (H) compared to their control mice. Male septic MMP7KO mice also had significantly higher BAL IL-10 (E) and lung tissue mRNA expression of IL-1β (F) compared to MMP7KO controls, while male septic WT mice had numerically higher BAL IL-10 and lung tissue mRNA expression of IL-1β compared to WT controls. Male septic mice did not differ from control mice across either genotype for BAL concentrations of IFN-γ (L) or BAL neutrophils (M). No differences between MMP7KO and WT genotypes were observed for any outcomes of lung inflammation in either treatment group. N = 3–11. [Statistical analysis: Kruskal-Wallis test with a Dunn’s multiple comparisons test]. Each point represents an individual animal. Horizontal line indicates median. Control = 5% dextrose + room air at 21% O_2_, BAL= bronchoalveolar lavage, TNF-α = tumor necrosis factor-α, IL-12p70 = interleukin-12p70, IL-6 = interleukin-6, IL-1β = interleukin-1β, CXCL-1 = C-X-C motif chemokine ligand 1, IFN-γ = interferon gamma, IL-10 = interleukin-10.
(TIF)

S8 Fig Alveolar-capillary barrier disruption induced by a septic insult [cecal slurry (CS; 1.6mg/g) + hyperoxia (HO; 95% O_2_)] in female MMP7KO mice and their wild-type littermates. Compared to control mice, septic WT showed significant increases in lung wet-to-dry weight ratios (A) and BAL protein (B), but not BAL IgM (C). Septic MMP7KO mice showed numerical increases in lung wet-to-dry weight ratios, BAL protein, and BAL IgM compared to MMP7KO controls. No differences were observed between WT and MMP7KO mice in either treatment group. N = 3–12. [Statistical analysis: Kruskal-Wallis test with a Dunn’s multiple comparisons test]. Each point represents an individual animal. Horizontal line indicates median. Control = 5% dextrose + room air at 21% O_2_, BAL= bronchoalveolar lavage.
(TIF)

S9 Fig Alveolar-capillary barrier disruption induced by a septic insult [cecal slurry (CS; 1.6mg/g) + hyperoxia (HO; 95% O_2_)] in male MMP7KO mice and their wild-type littermates. Compared to control mice, septic MMP7KO mice showed significant increases in lung wet-to-dry weight ratios (A). No differences were observed between septic and control mice across either genotype for BAL protein (B) or BAL IgM concentration (C). Similarly, no differences were observed between WT and MMP7KO mice in either treatment group. N = 3–10. [Statistical analysis: Kruskal-Wallis test with a Dunn’s multiple comparisons test]. Each point represents an individual animal. Horizontal line indicates median. Control = 5% dextrose + room air at 21% O_2_, BAL= bronchoalveolar lavage.
(TIF)

S10 Fig Histological Lung Injury induced by a septic insult [cecal slurry (CS; 1.6mg/g) + hyperoxia (HO; 95% O_2_)] in female MMP7KO mice and their wild-type littermates. Representative whole lung sections in female mice used for histology scoring (A). Lungs were stained with H&E and 20x images of the entire section scanned by the Vanderbilt University Medical Center Digital Histology Shared Resource using the Leica SCN400 Slide Scanner. Due to the low number of female control mice in the histology cohort, it is unclear whether female septic mice differed from female control mice with respect to total lung injury score (B) in either genotype. No differences were observed between WT and MMP7KO septic mice. Individual parameters of lung histology injury score are presented as a table (C). N = 1–5. [Statistical analysis: Kruskal-Wallis test with a Dunn’s multiple comparisons test]. Each point represents an individual animal. Horizontal line indicates median. Control = 5% dextrose + room air at 21% O_2_. RBC = red blood cell, IQR = interquartile range.
(TIF)

S11 Fig Histological Lung Injury induced by a septic insult [cecal slurry (CS; 1.6mg/g) + hyperoxia (HO; 95% O_2_)] in male MMP7KO mice and their wild-type littermates. Representative whole lung sections in male mice used for histology scoring (A). Lungs were stained with H&E and 20x images of the entire section scanned by the Vanderbilt University Medical Center Digital Histology Shared Resource using the Leica SCN400 Slide Scanner. Male septic mice (both WT and MMP7KO) showed numerical increases in total lung injury score compared to control mice (B). However, no differences were observed between the genotypes for either treatment group. Individual parameters of lung histology injury score are presented as a table (C). N = 2–5. [Statistical analysis: Kruskal-Wallis test with a Dunn’s multiple comparisons test]. Each point represents an individual animal. Horizontal line indicates median. Control = 5% dextrose + room air at 21% O_2_. RBC = red blood cell, IQR = interquartile range.
(TIF)

S12 Fig Kidney Dysfunction and Injury induced by a septic insult [cecal slurry (CS; 1.6mg/g) + hyperoxia (HO; 95% O_2_)] in female MMP7KO mice and their wild-type littermates. No differences were observed between septic MMP7KO and WT mice for plasma urea nitrogen concentration (A), kidney tissue mRNA levels of IL-1β (B), IL-6 (C), CXCL-1 (D), NGAL (E), or KIM-1 (H), protein levels of NGAL (G) or KIM-1 (J). Septic WT mice showed significantly higher plasma urea nitrogen concentrations, kidney tissue mRNA expression of IL-1β, CXCL-1, NGAL, and KIM-1. Septic WT mice also showed numerically higher kidney tissue mRNA expression of IL-1β and KIM-1 protein. Although MMP7KO mice showed similar numerical increases they were not significant compared to MMP7KO controls. No differences were observed between control WT and MMP7KO mice. Representative western blots for female mice are displayed for NGAL (F) and KIM-1 (I) relative to β-actin. N = 3–10. [Statistical analysis: Kruskal-Wallis test with a Dunn’s multiple comparisons test]. Each point represents an individual animal. Horizontal line indicates combined median. Control = 5% dextrose + room air at 21% O_2_. NGAL = neutrophil gelatinase-associated lipocalin, KIM-1 = kidney injury marker-1, IL-6 = interleukin-6, IL-1β = interleukin-1β, CXCL-1 = C-X-C motif chemokine ligand 1.
(TIF)

S13 Fig Kidney Dysfunction and Injury induced by a septic insult [cecal slurry (CS; 1.6mg/g) + hyperoxia (HO; 95% O_2_)] in male MMP7KO mice and their wild-type littermates. No differences were observed between septic MMP7KO and WT mice for plasma urea nitrogen concentration (A), kidney tissue mRNA levels of IL-1β (B), IL-6 (C), CXCL-1 (D), NGAL (E), or KIM-1 (H), protein levels of NGAL (G) or KIM-1 (J). Septic WT mice showed significantly higher plasma urea nitrogen concentrations, kidney tissue mRNA expression of IL-1β, CXCL-1, and NGAL. Septic WT mice also showed numerically higher kidney tissue mRNA expression of IL-6 and KIM-1 protein. Although MMP7KO mice showed similar numerical increases they were not significant compared to MMP7KO controls. No differences were observed between control WT and MMP7KO mice. Representative western blots for male mice are displayed for NGAL (F) and KIM-1 (I) relative to β-actin. N = 3–10. [Statistical analysis: Kruskal-Wallis test with a Dunn’s multiple comparisons test]. Each point represents an individual. Horizontal line indicates median. Control = 5% dextrose + room air at 21% O_2_. NGAL = neutrophil gelatinase-associated lipocalin, KIM-1 = kidney injury marker-1, IL-6 = interleukin-6, IL-1β = interleukin-1β, CXCL-1 = C-X-C motif chemokine ligand 1.
(TIF)

S14 Fig Lung and Kidney Matrix Metalloproteinase Expression induced by a septic insult [cecal slurry (CS; 1.6mg/g) + hyperoxia (HO; 95% O_2_)] in female MMP7KO mice and their wild-type littermates. WT Septic mice had significantly higher MMP2 (A) and MMP9 (B), as well as numerically higher MMP7 (C) mRNA expression in lung tissue. WT septic mice also had significantly higher MMP9 (E) mRNA as well as numerically higher MMP7 (p=0.2086; F) in kidney tissue. Septic mice (WT or MMP7KO) did not differ from control mice with respect to kidney mRNA expression of MMP2 (D). Compared to control mice female septic MMP7KO mice did not have significant differences across any of the MMPs measured compared to control MMP7KO mice. Female MMP7KO septic mice had significantly lower lung and kidney MMP7 mRNA expression compared to septic WT mice, but did not differ with regards to MMP9 or MMP2 in either organ. Although no differences were observed between genotypes for control mice, control MMP7KO mice had numerically higher lung MMP2 and kidney MMP9 compared to control WT mice. N = 3–13. [Statistical analysis: Kruskal-Wallis test with a Dunn’s multiple comparisons test]. Each point represents an individual animal. Horizontal line indicates median. Control = 5% dextrose + room air at 21% O_2_. MMP7 = matrix metalloproteinase-7, MMP9 = matrix metalloproteinase-9, MMP2 = matrix metalloproteinase-2.
(TIF)

S15 Fig Lung and Kidney Matrix Metalloproteinase Expression induced by a septic insult [cecal slurry (CS; 1.6mg/g) + hyperoxia (HO; 95% O_2_)] in male MMP7KO mice and their wild-type littermates. No differences were observed for lung tissue mRNA expression of MMP2 (A), MMP9 (B), or MMP7 (C) between male septic WT and MMP7KO mice. Although septic MMP7KO mice had significantly lower kidney mRNA expression of MMP7 (F) compared to septic WT mice, there were no differences across kidney mRNA expression of MMP2 (D) or MMP9 (E). Septic MMP7KO mice also had significantly higher lung mRNA expression of MMP9 compared to control MMP7KO mice, while WT had numerically higher MMP9 compared to WT controls. Septic mice (both WT and MMP7KO) showed numerical increases in kidney MMP9 mRNA expression compared to control mice. No differences were observed between septic mice and control for MMP2 mRNA expression in the lung or kidney. N = 3–8. [Statistical analysis: Kruskal-Wallis test with a Dunn’s multiple comparisons test]. Each point represents an individual animal. Horizontal line indicates median. Control = 5% dextrose + room air at 21% O_2_. MMP7 = matrix metalloproteinase-7, MMP9 = matrix metalloproteinase-9, MMP2 = matrix metalloproteinase-2.
(TIF)

S1 Data Complete data set. (XLSX)

S1 Raw Images Uncut western blot images. (PDF)

## Abbreviations

VUMC: Vanderbilt University Medical Center
ARDS: acute respiratory distress syndrome
MMP: matrix metalloproteinase
ALI: acute lung injury
KO: knockout
WT: wild-type littermate
CS: cecal slurry
HO: hyperoxia
FiO2: fraction of inspired oxygen
BAL: bronchoalveolar lavage
NGAL: neutrophil gelatinase-associated lipocalin
KIM-1: kidney injury molecule 1
ICU: intensive care unit
LPS: lipopolysaccharide
IP: intraperitoneal injection
SQ: subcutaneous injection
K2EDTA: dipotassium ethylenediaminetetraacectic acid
CFU: colony forming units
PFA: paraformaldehyde
IL-1β: interleukin-1β
IL-6: interleukin-6
IL-10: interleukin-10
IL-12p70: interleukin-12p70
TNF-α: tumor necrosis factor α
CXCL-1: C-X-C motif chemokine ligand 1
IFN-γ: interferon-γ
IgM: immunoglobulin M
RIPA: radioimmunoprecipitation assay
RBC: red blood cell
IQR: interquartile range
