# Supplementary figures and images for: Matrix metalloproteinase-7 is dispensable in a mouse model of sepsis-induced acute lung injury
Source: PLoS One. 2025 May 8;20(5):e0321349. doi: 10.1371/journal.pone.0321349 (PMC12061409; doi:10.1371/journal.pone.0321349)

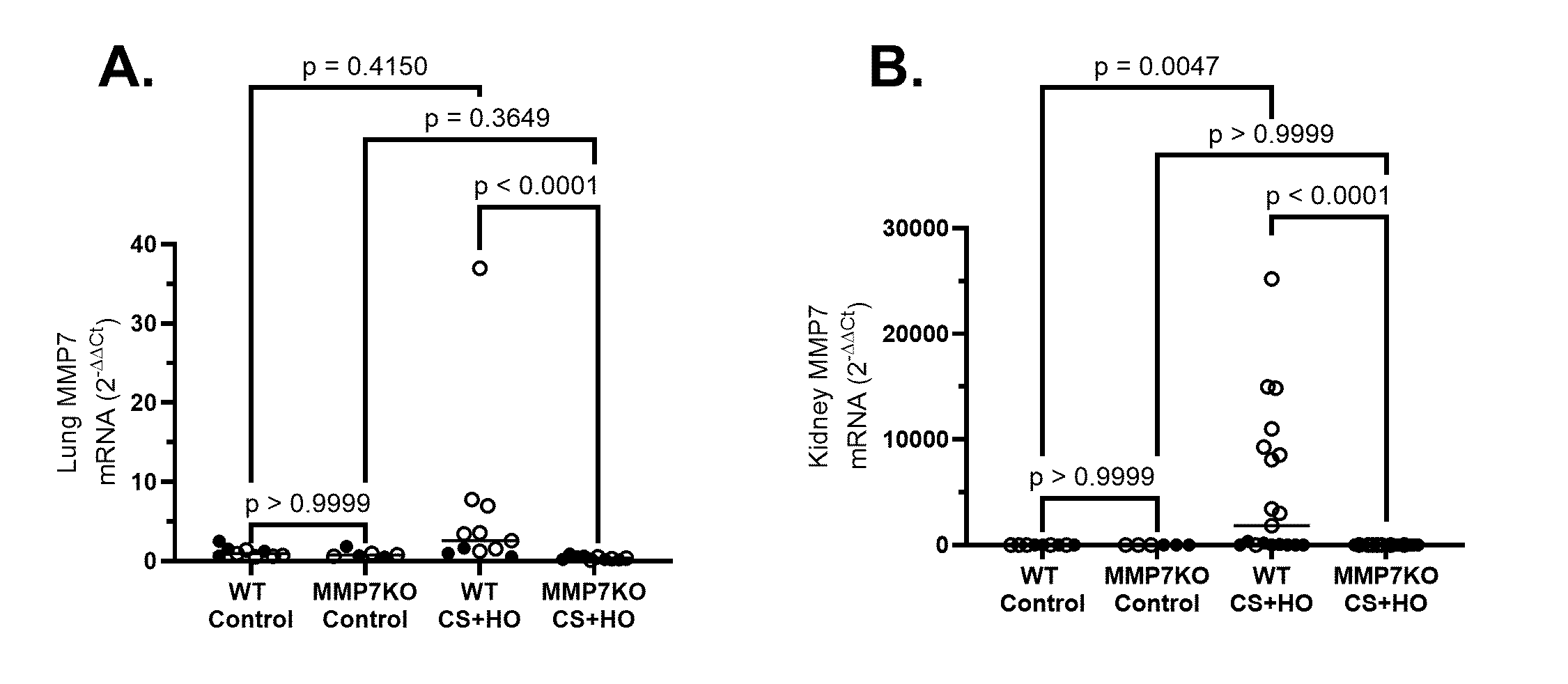

Supplement: S1 Fig — WT Septic mice had significantly higher lung (A) and kidney (B) MMP7 mRNA expression compared to WT control mice. No differences were observed between genotypes for control mice. N = 6–18. [Statistical analysis: Kruskal-Wallis test with a Dunn’s multiple comparisons test]. Each point represents an individual male (solid circle) or female (open circle) animal. Horizontal line indicates combined median of male and female animals. Control = 5% dextrose + room air at 21% O2. MMP7 = matrix metalloproteinase-7. (TIF) [file pone.0321349.s001.tif]

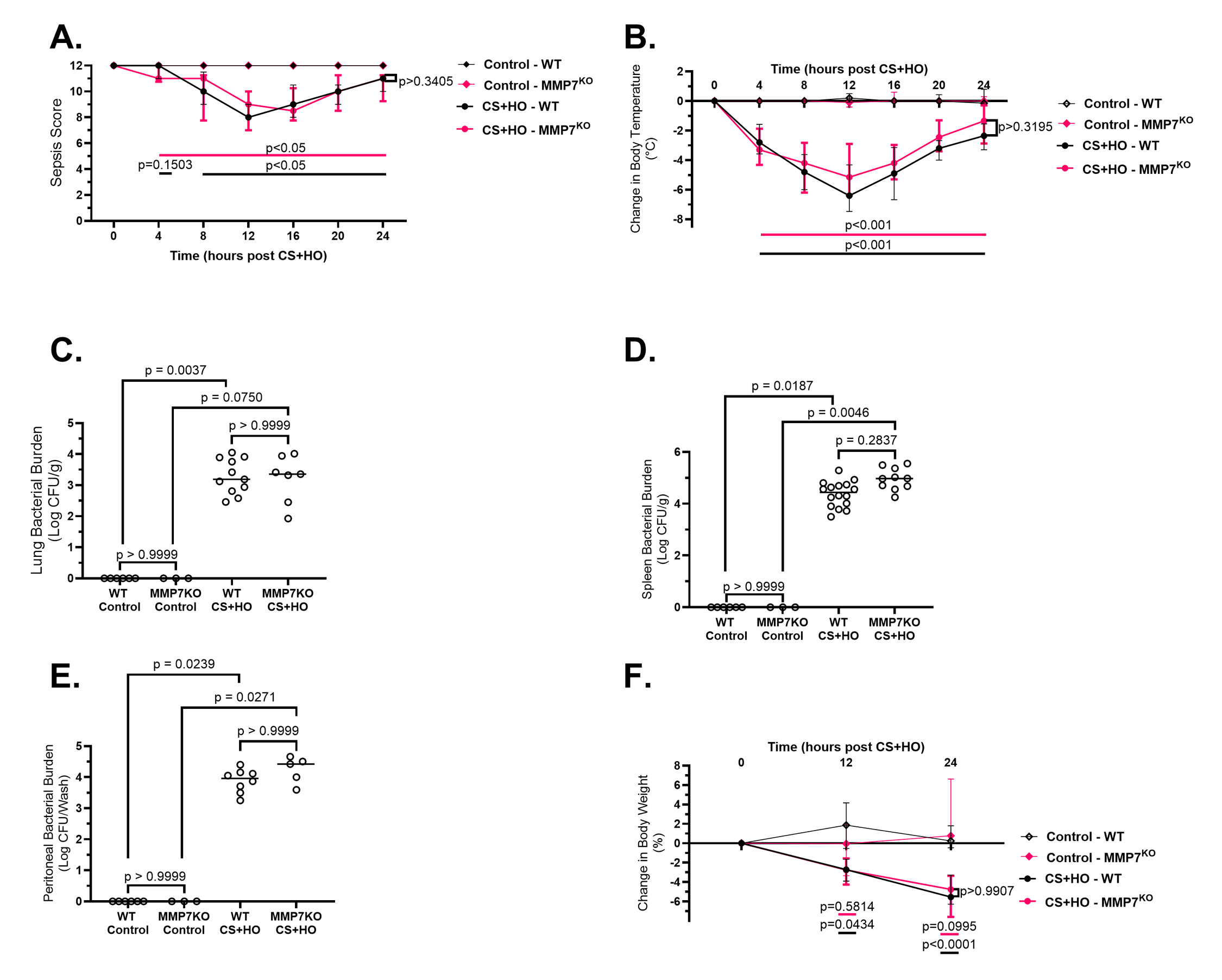

Supplement: S2 Fig — Septic mice (in both WT and MMP7KO) showed significantly greater illness severity (A) and loss of body temperature (B) at all timepoints after 8 hours post-CS+HO, as well as spleen bacterial burden (C), peritoneal bacterial burden (D), and lung bacterial burden (E) at 24-hours post CS+HO compared to control mice. WT septic mice also showed significantly greater weight loss compared to WT control mice at all 12-hours and 24-hours post CS+HO (F). MMP7KO septic mice had numerically greater weight loss at 12-hours and 24-hours post CS+HO compared to MMP7KO control mice. No differences were observed across the genotypes for septic mice any of the measured outcomes. N = 3–13. [Statistical analysis: Repeated Two-way ANOVA (A, B, F); Kruskal-Wallis test with a Dunn’s multiple comparisons test (C-E)]. Each point represents either the median (A, B, F) or an individual animal with a horizontal line indicating median (D-F). Error bars indicate interquartile range, while horizontal lines represent statistical comparisons between septic WT (black) or MMP7KO (pink) and their respective controls (A, B, F). Control = 5% dextrose + room air at 21% O2. (TIF) [file pone.0321349.s002.tif]

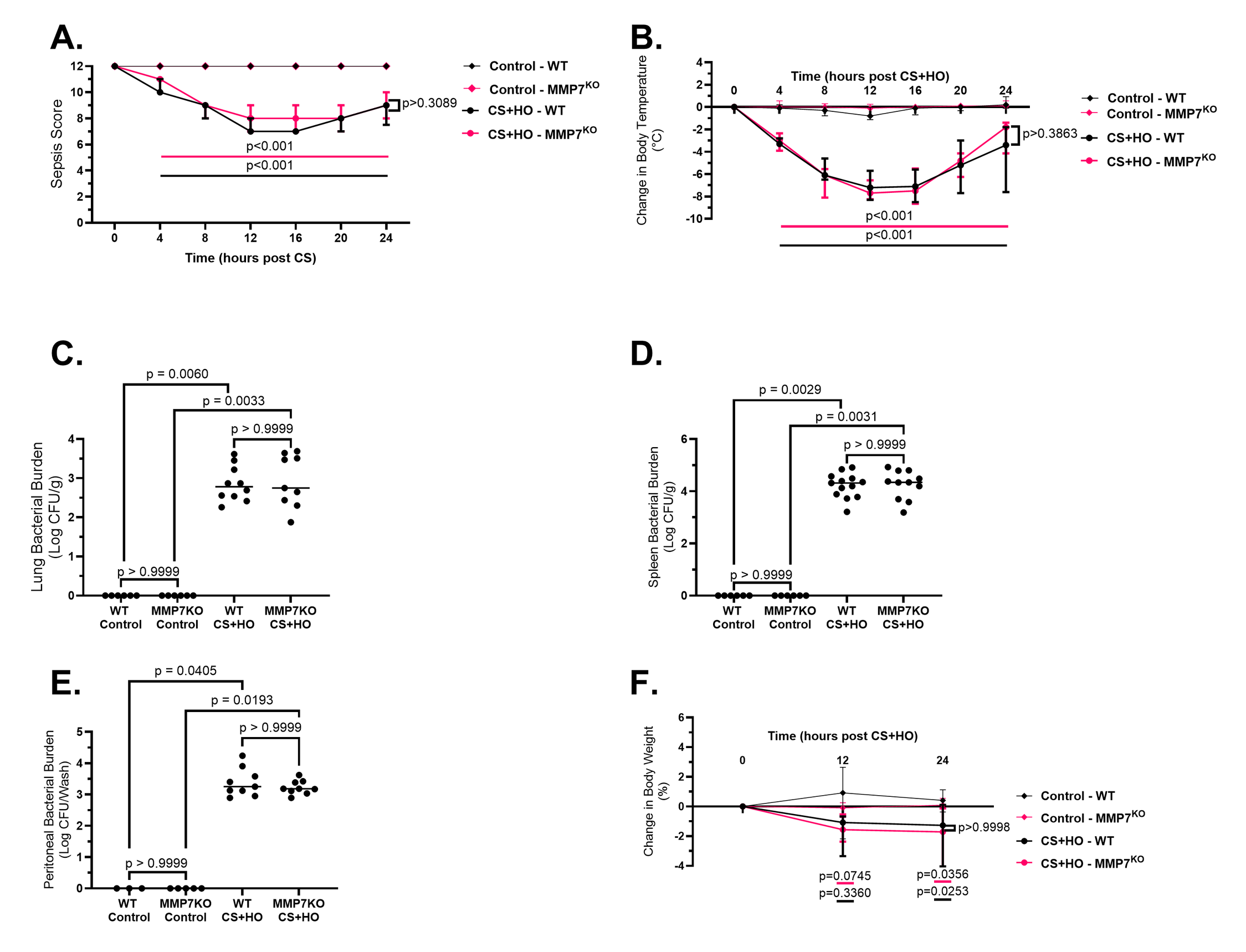

Supplement: S3 Fig — Septic mice (in both WT and MMP7KO) showed significantly greater illness severity (A) and loss of body temperature (B) at all timepoints after 8 hours post-CS+HO, as well as spleen bacterial burden (C), peritoneal bacterial burden (D), and lung bacterial burden (E) at 24-hours post CS+HO compared to control mice. WT septic mice also showed significantly greater weight loss at 24-hours post CS+HO (F) and numerically higher weight loss at 12 hours post CS+HO compared to control WT mice. MMP7KO septic mice had numerically greater weight loss at 12-hours, with significantly higher weight loss at 24-hours post CS+HO compared to MMP7KO control mice. No differences were observed between the genotypes for septic mice across any of the measured outcomes. N = 3–13. [Statistical analysis: Repeated Two-way ANOVA (A, B, F); Kruskal-Wallis test with a Dunn’s multiple comparisons test (C-E)]. Each point represents either the median (A, B, F) or an individual animal with a horizontal line indicating median (D-F). Error bars indicate interquartile range, while horizontal lines represent statistical comparisons between septic WT (black) or MMP7KO (pink) and their respective controls (A, B, F). Control = 5% dextrose + room air at 21% O2. (TIF) [file pone.0321349.s003.tif]

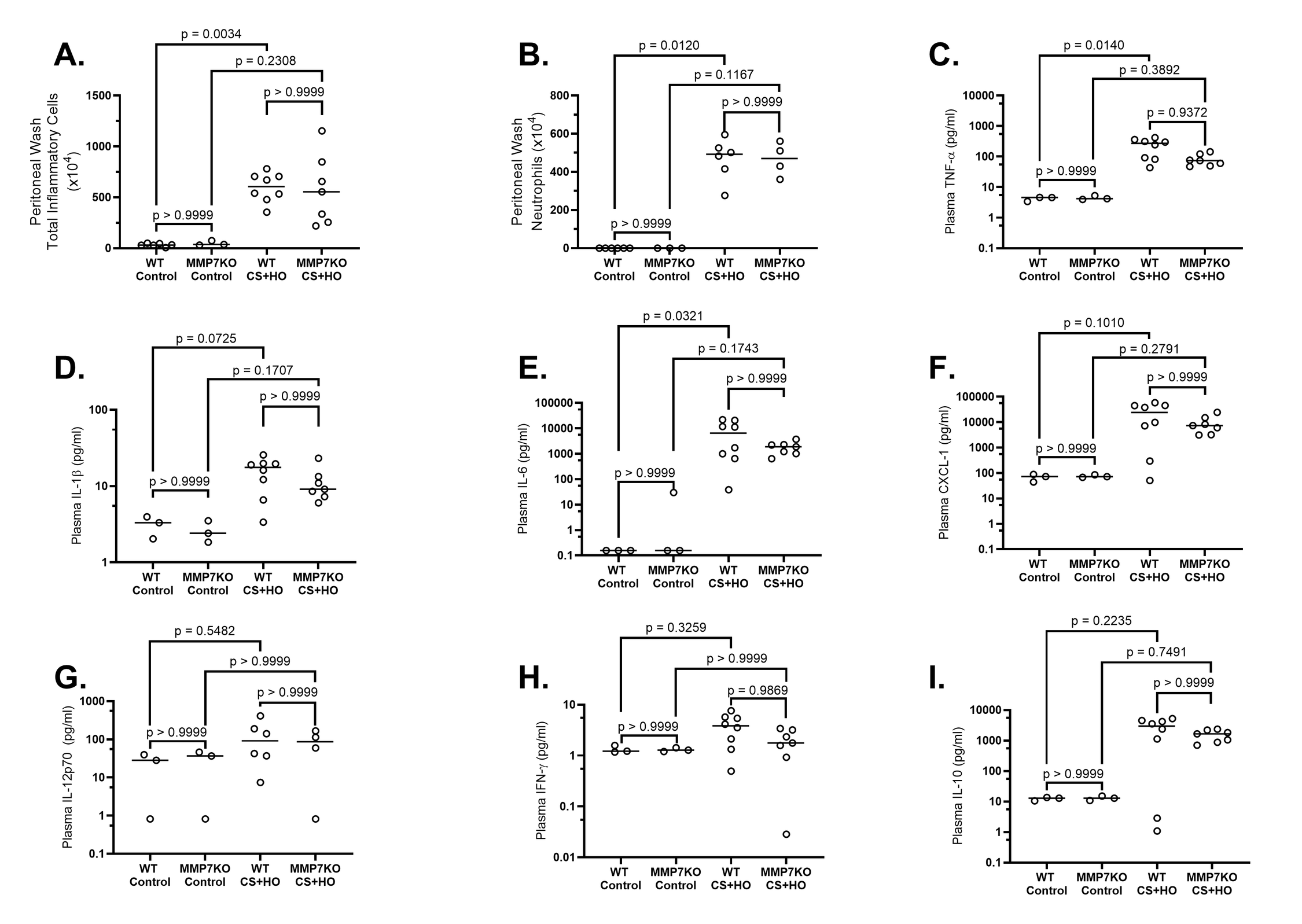

Supplement: S4 Fig — Septic WT mice had significantly higher peritoneal wash inflammatory cell counts (A), peritoneal wash neutrophil counts (B), and plasma concentrations of concentrations of TNF-α (C), and IL-6 (E) compared to their control mice. Female septic WT mice also had numerically higher plasma concentrations of IL-1β (D), CXCL-1 (F), IL-12p70 (G), IFN-γ (H), and IL-10 (I). Although female septic MMP7KO mice showed similar numerical increases across all pro-inflammatory cytokines, they were not significantly different compared to MMP7KO controls. No differences were observed between MMP7KO and WT mice in either treatment group. N = 3–8. [Statistical analysis: Kruskal-Wallis test with a Dunn’s multiple comparisons test]. Each point represents an individual animal. Horizontal line indicates median. Control = 5% dextrose + room air at 21% O2. TNF-α = tumor necrosis factor-α, IL-12p70 = interleukin-12p70, IL-6 = interleukin-6, IL-1β = interleukin-1β, CXCL-1 = C-X-C motif chemokine ligand 1, IFN-γ = interferon gamma, IL-10 = interleukin-10. (TIF) [file pone.0321349.s004.tif]

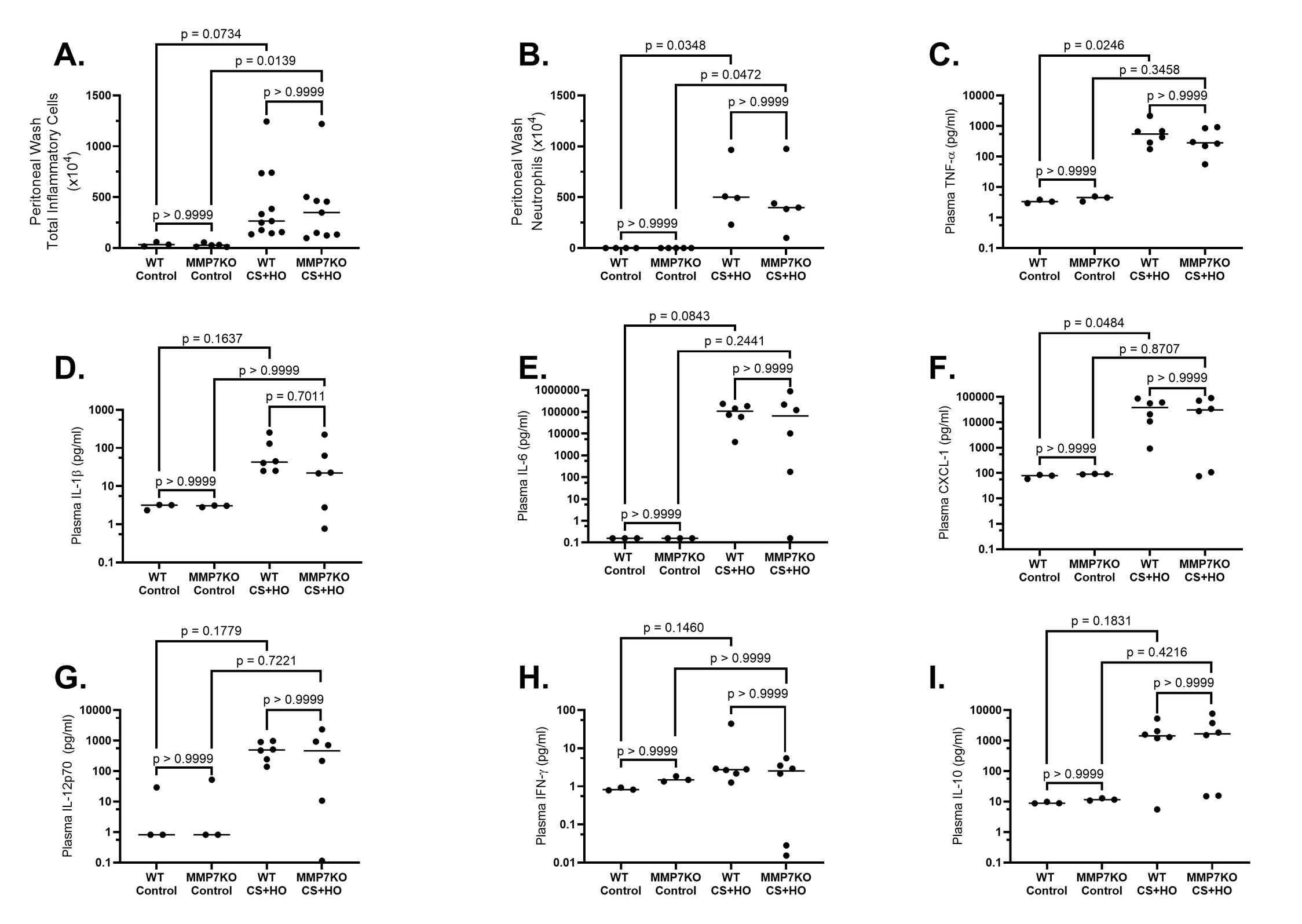

Supplement: S5 Fig — Septic WT mice had numerically higher peritoneal wash inflammatory cell counts (A), peritoneal wash neutrophil counts (B), and plasma concentrations of concentrations of TNF-α (C), and CXCL-1 (F) compared to their control mice. Male septic WT mice also had numerically higher plasma concentrations of IL-1β (D), IL-6 (E), IL-12p70 (G), IFN-γ (H), and IL-10 (I). Male septic MMP7KO mice showed significantly higher peritoneal inflammatory cell counts compared to control MMP7KO mice. Although male septic MMP7KO mice had similar numerical increases across all pro-inflammatory cytokines as WT, they were not significantly different compared to MMP7KO controls. No differences were observed between MMP7KO and WT mice in either treatment group. N = 3–11. [Statistical analysis: Kruskal-Wallis test with a Dunn’s multiple comparisons test]. Each point represents an individual animal. Horizontal line indicates median. Control = 5% dextrose + room air at 21% O2. TNF-α = tumor necrosis factor-α, IL-12p70 = interleukin-12p70, IL-6 = interleukin-6, IL-1β = interleukin-1β, CXCL-1 = C-X-C motif chemokine ligand 1, IFN-γ = interferon gamma, IL-10 = interleukin-10. (TIF) [file pone.0321349.s005.tif]

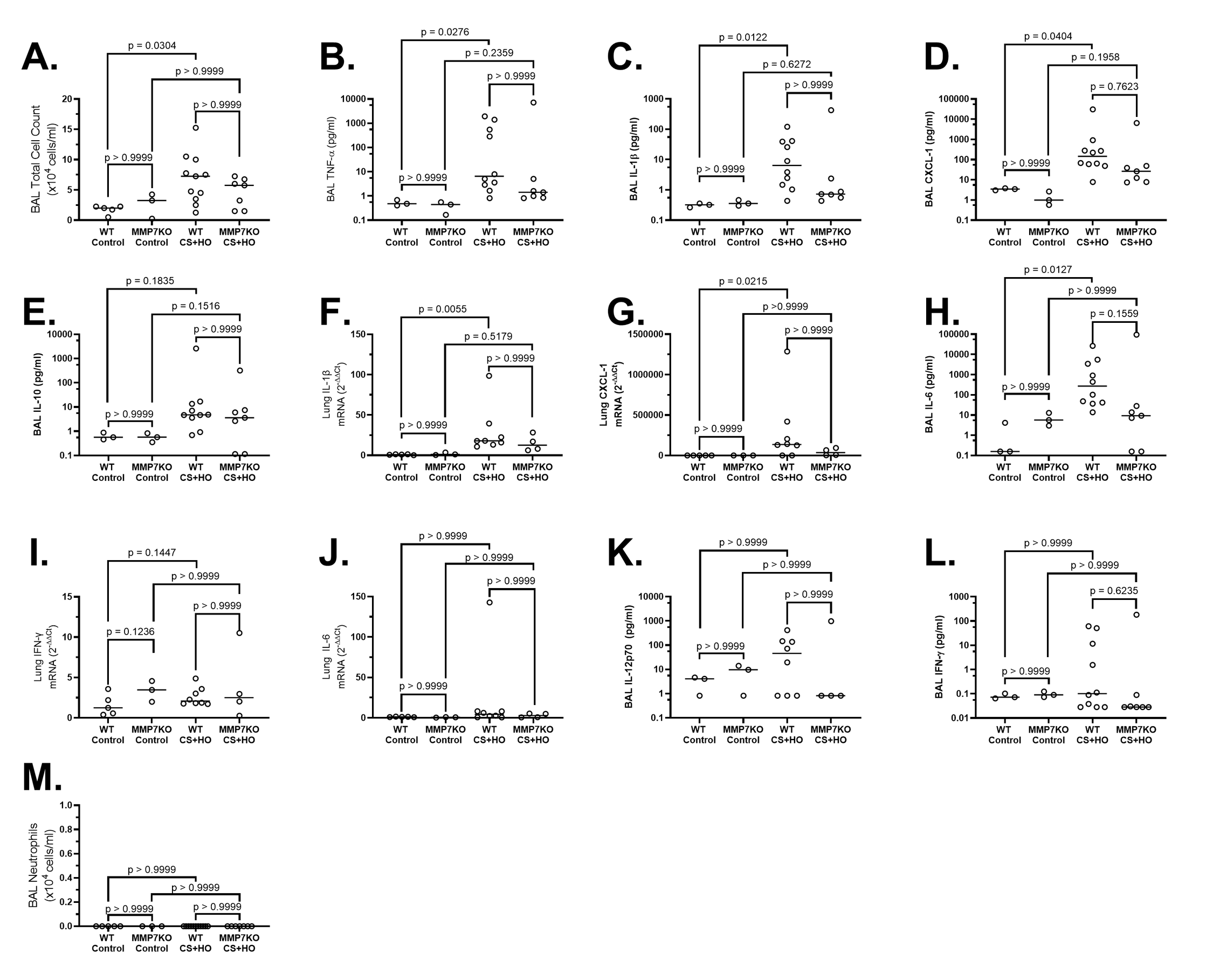

Supplement: S6 Fig — Septic WT mice had significantly higher BAL total cell counts (A), BAL concentrations of TNF-α (B), IL-1β (C), CXCL-1 (D), and IL-6 (H), as well as lung tissue mRNA expression of IL-1b (F) and CXCL-1 (G) compared to their control mice. Septic WT also had numerically higher BAL IL-10 (E) and lung tissue mRNA expression of IFN-γ (I) compared to WT controls. Although septic MMP7KO mice showed similar numerical increases they were not significantly different compared to MMP7KO controls. Septic mice did not differ from control mice across either genotype for lung tissue mRNA expression of IFN-γ (I), IL-6 (J), BAL IL-12p70 (K), IFN-γ (L), or BAL neutrophils (M). No differences between MMP7KO and WT genotypes were observed for any outcomes of lung inflammation in either treatment group. N = 3–11. [Statistical analysis: Kruskal-Wallis test with a Dunn’s multiple comparisons test]. Each point represents an individual animal. Horizontal line indicates median. Control = 5% dextrose + room air at 21% O2, BAL= bronchoalveolar lavage, TNF-α = tumor necrosis factor-α, IL-12p70 = interleukin-12p70, IL-6 = interleukin-6, IL-1β = interleukin-1β, CXCL-1 = C-X-C motif chemokine ligand 1, IFN-γ = interferon gamma, IL-10 = interleukin-10. (TIF) [file pone.0321349.s006.tif]

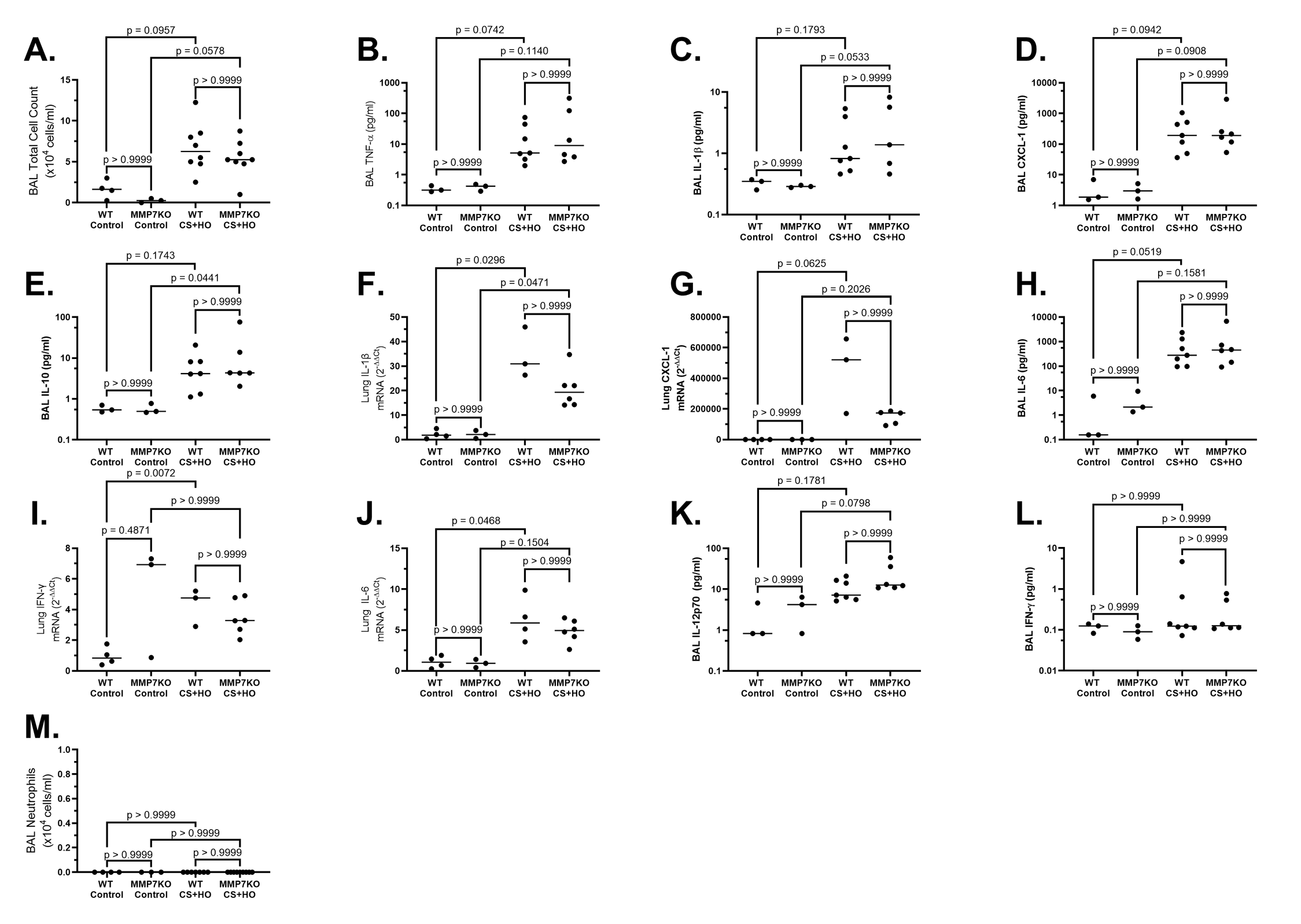

Supplement: S7 Fig — Male septic (both WT and MMP7KO) mice had numerically higher BAL total cell counts (A), BAL concentrations of TNF-α (B), IL-1β (C), CXCL-1 (D), IL-6 (H), and IL-12p70 (K) as well as lung tissue mRNA expression of CXCL-1 (G) and IL-6 (H) compared to their control mice. Male septic MMP7KO mice also had significantly higher BAL IL-10 (E) and lung tissue mRNA expression of IL-1β (F) compared to MMP7KO controls, while male septic WT mice had numerically higher BAL IL-10 and lung tissue mRNA expression of IL-1β compared to WT controls. Male septic mice did not differ from control mice across either genotype for BAL concentrations of IFN-γ (L) or BAL neutrophils (M). No differences between MMP7KO and WT genotypes were observed for any outcomes of lung inflammation in either treatment group. N = 3–11. [Statistical analysis: Kruskal-Wallis test with a Dunn’s multiple comparisons test]. Each point represents an individual animal. Horizontal line indicates median. Control = 5% dextrose + room air at 21% O2, BAL= bronchoalveolar lavage, TNF-α = tumor necrosis factor-α, IL-12p70 = interleukin-12p70, IL-6 = interleukin-6, IL-1β = interleukin-1β, CXCL-1 = C-X-C motif chemokine ligand 1, IFN-γ = interferon gamma, IL-10 = interleukin-10. (TIF) [file pone.0321349.s007.tif]

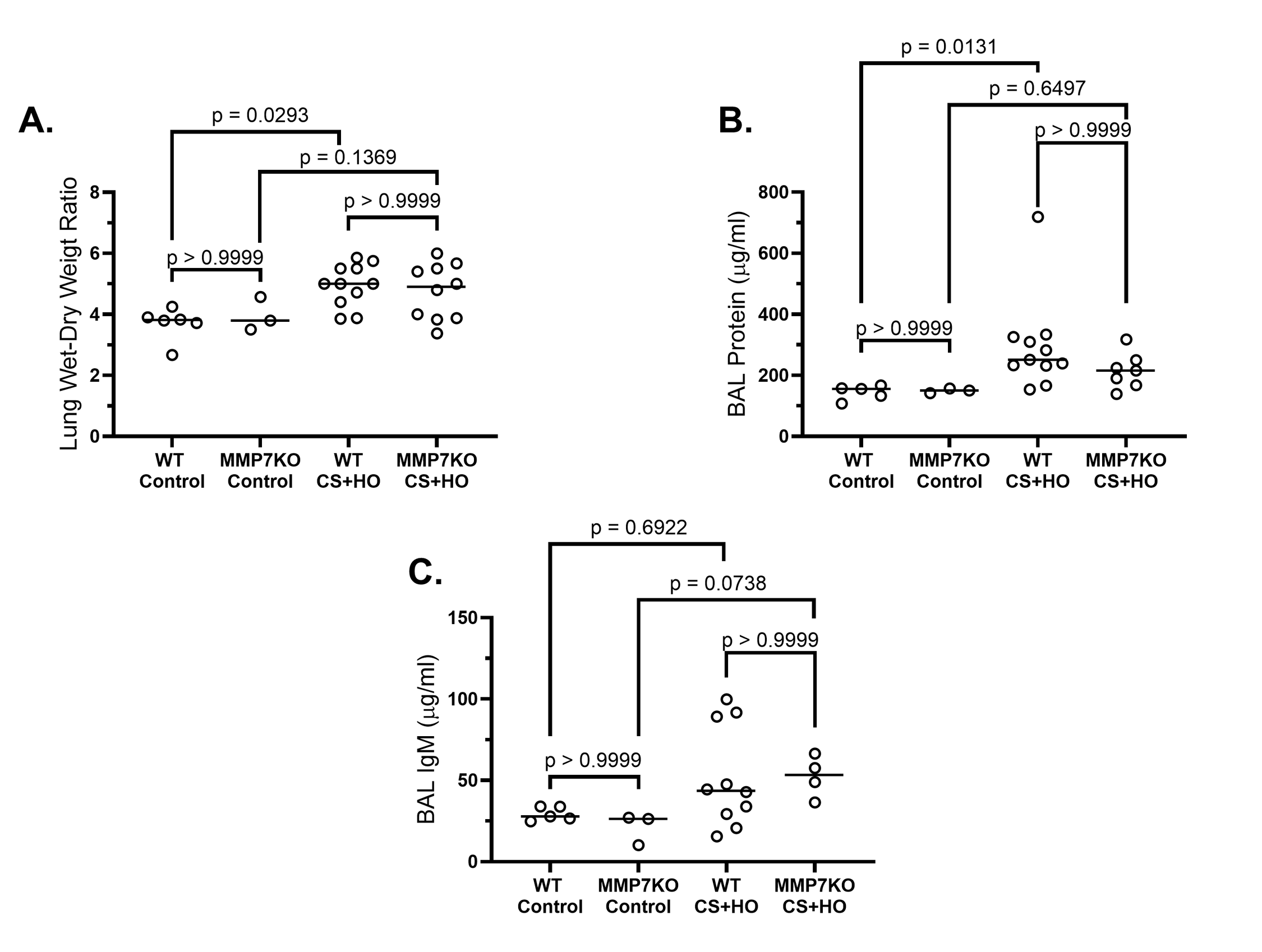

Supplement: S8 Fig — Compared to control mice, septic WT showed significant increases in lung wet-to-dry weight ratios (A) and BAL protein (B), but not BAL IgM (C). Septic MMP7KO mice showed numerical increases in lung wet-to-dry weight ratios, BAL protein, and BAL IgM compared to MMP7KO controls. No differences were observed between WT and MMP7KO mice in either treatment group. N = 3–12. [Statistical analysis: Kruskal-Wallis test with a Dunn’s multiple comparisons test]. Each point represents an individual animal. Horizontal line indicates median. Control = 5% dextrose + room air at 21% O2, BAL= bronchoalveolar lavage. (TIF) [file pone.0321349.s008.tif]

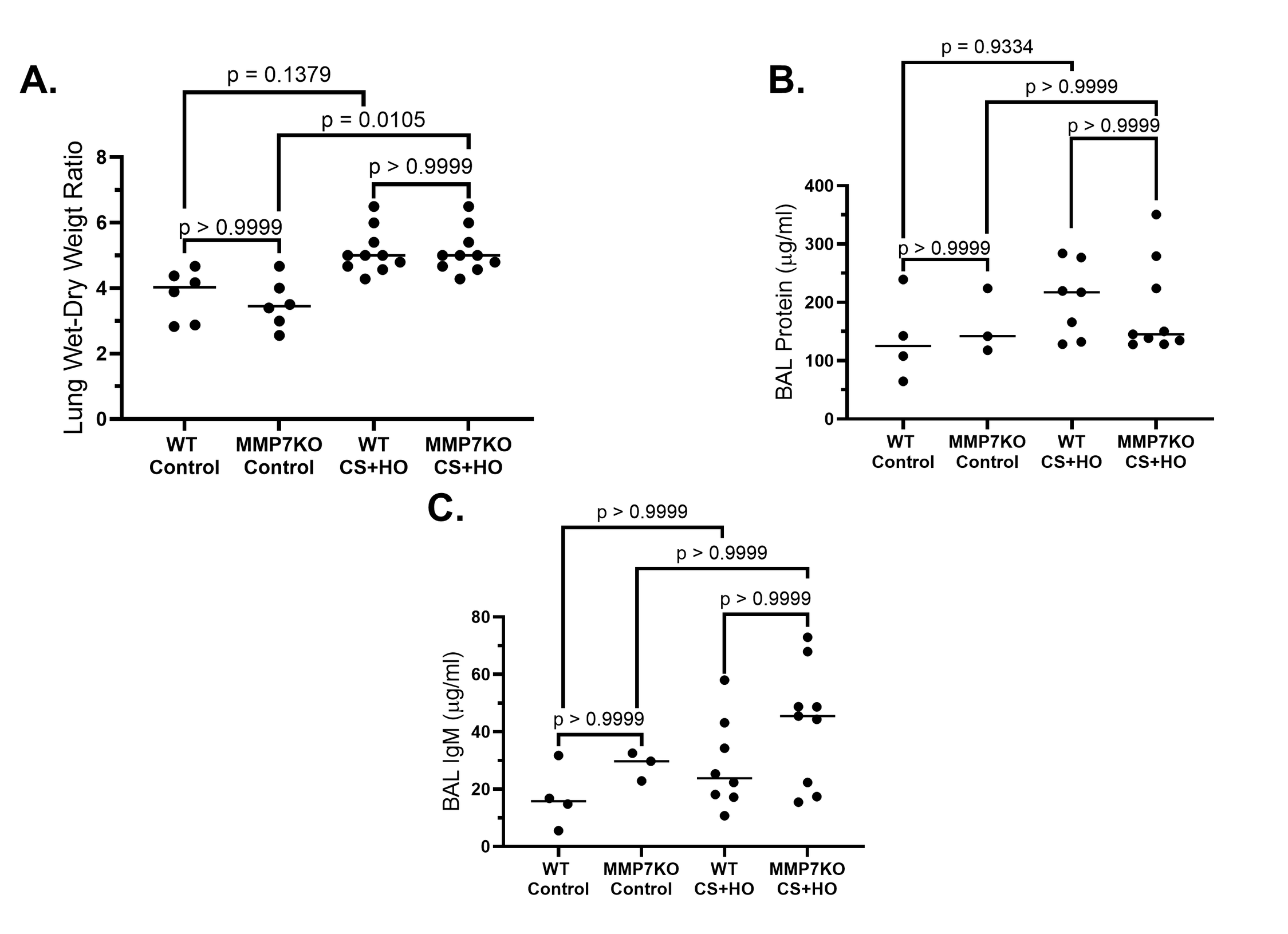

Supplement: S9 Fig — Compared to control mice, septic MMP7KO mice showed significant increases in lung wet-to-dry weight ratios (A). No differences were observed between septic and control mice across either genotype for BAL protein (B) or BAL IgM concentration (C). Similarly, no differences were observed between WT and MMP7KO mice in either treatment group. N = 3–10. [Statistical analysis: Kruskal-Wallis test with a Dunn’s multiple comparisons test]. Each point represents an individual animal. Horizontal line indicates median. Control = 5% dextrose + room air at 21% O2, BAL= bronchoalveolar lavage. (TIF) [file pone.0321349.s009.tif]

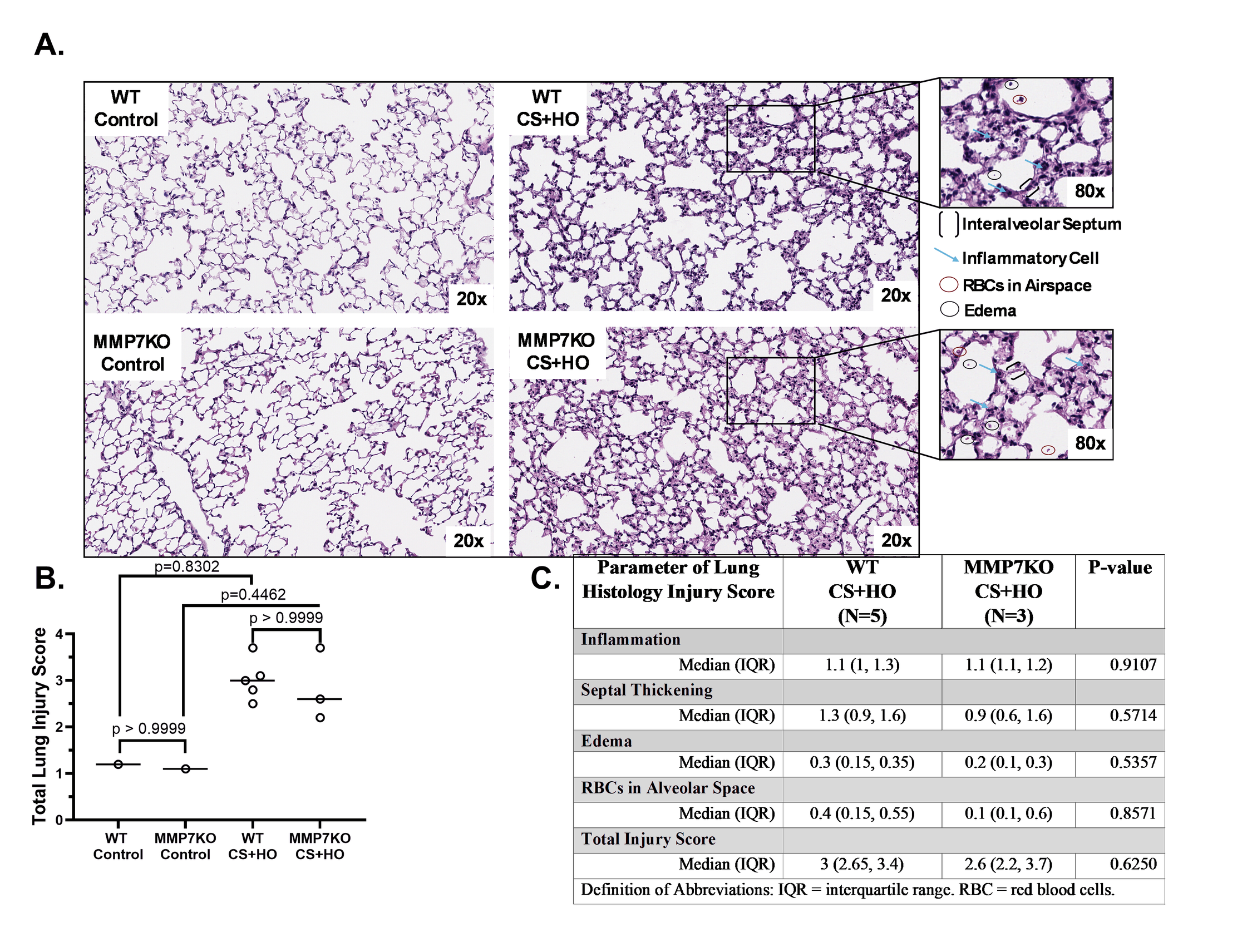

Supplement: S10 Fig — Representative whole lung sections in female mice used for histology scoring (A). Lungs were stained with H&E and 20x images of the entire section scanned by the Vanderbilt University Medical Center Digital Histology Shared Resource using the Leica SCN400 Slide Scanner. Due to the low number of female control mice in the histology cohort, it is unclear whether female septic mice differed from female control mice with respect to total lung injury score (B) in either genotype. No differences were observed between WT and MMP7KO septic mice. Individual parameters of lung histology injury score are presented as a table (C). N = 1–5. [Statistical analysis: Kruskal-Wallis test with a Dunn’s multiple comparisons test]. Each point represents an individual animal. Horizontal line indicates median. Control = 5% dextrose + room air at 21% O2. RBC = red blood cell, IQR = interquartile range. (TIF) [file pone.0321349.s010.tif]

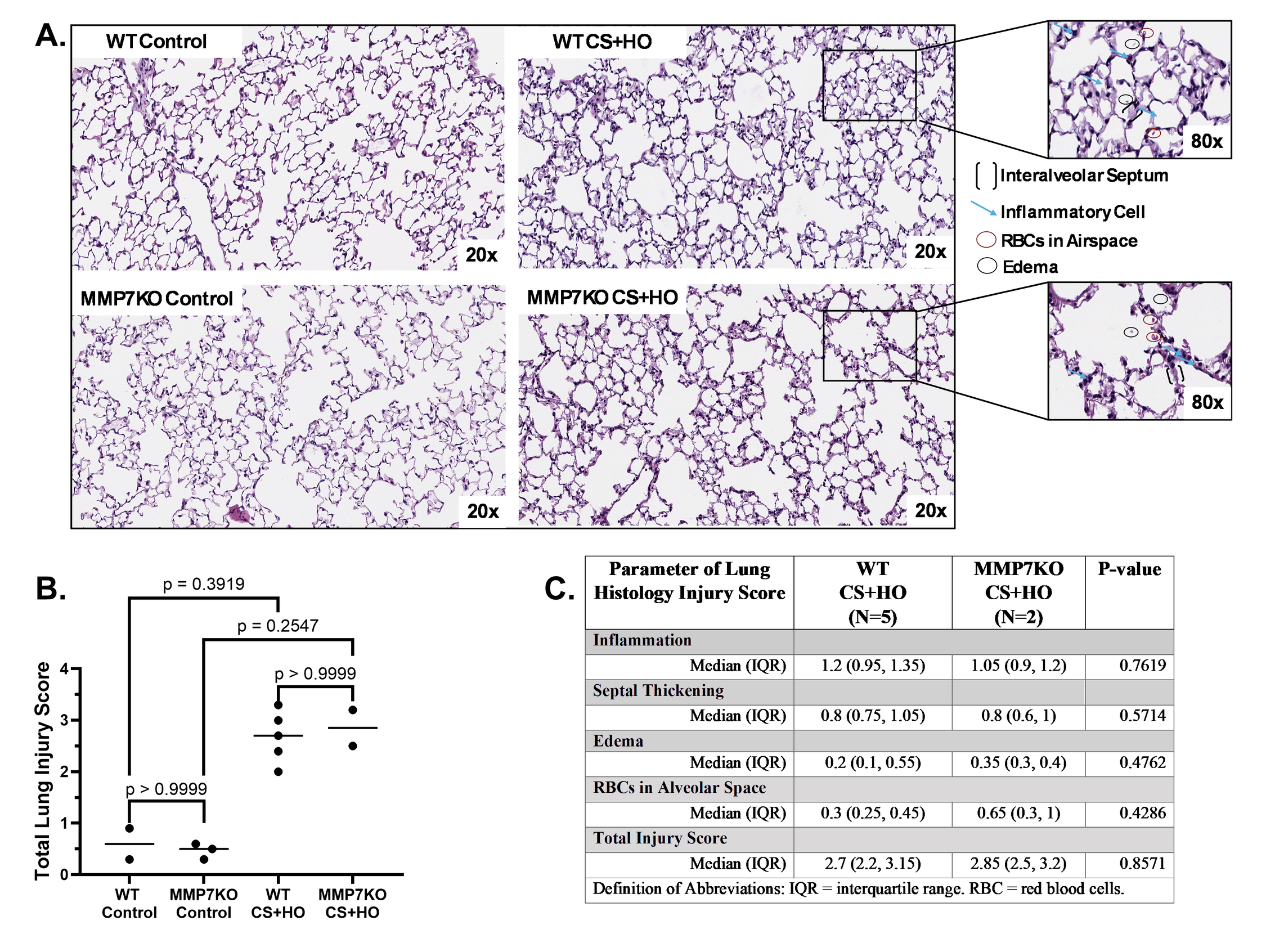

Supplement: S11 Fig — Representative whole lung sections in male mice used for histology scoring (A). Lungs were stained with H&E and 20x images of the entire section scanned by the Vanderbilt University Medical Center Digital Histology Shared Resource using the Leica SCN400 Slide Scanner. Male septic mice (both WT and MMP7KO) showed numerical increases in total lung injury score compared to control mice (B). However, no differences were observed between the genotypes for either treatment group. Individual parameters of lung histology injury score are presented as a table (C). N = 2–5. [Statistical analysis: Kruskal-Wallis test with a Dunn’s multiple comparisons test]. Each point represents an individual animal. Horizontal line indicates median. Control = 5% dextrose + room air at 21% O2. RBC = red blood cell, IQR = interquartile range. (TIF) [file pone.0321349.s011.tif]

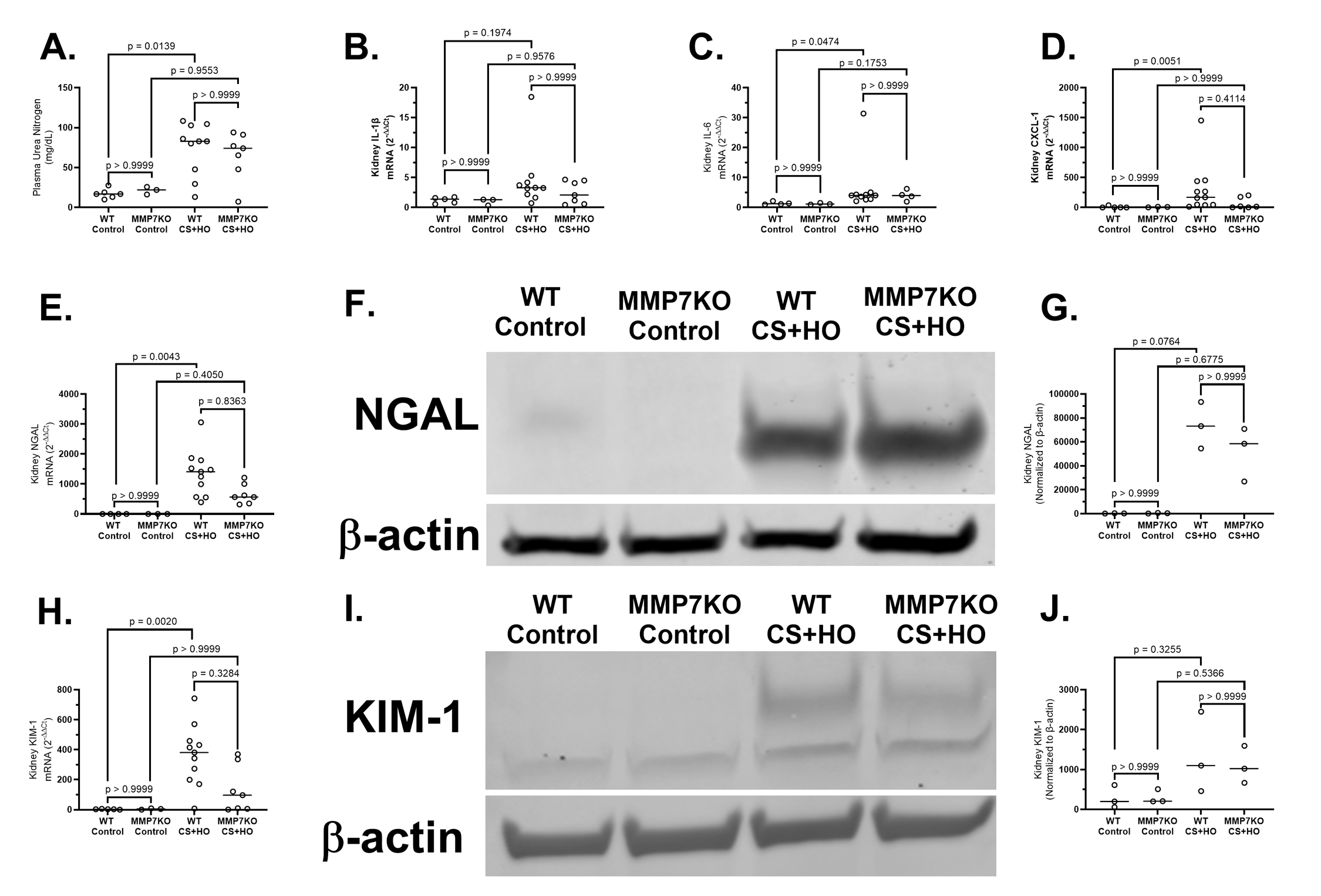

Supplement: S12 Fig — No differences were observed between septic MMP7KO and WT mice for plasma urea nitrogen concentration (A), kidney tissue mRNA levels of IL-1β (B), IL-6 (C), CXCL-1 (D), NGAL (E), or KIM-1 (H), protein levels of NGAL (G) or KIM-1 (J). Septic WT mice showed significantly higher plasma urea nitrogen concentrations, kidney tissue mRNA expression of IL-1β, CXCL-1, NGAL, and KIM-1. Septic WT mice also showed numerically higher kidney tissue mRNA expression of IL-1β and KIM-1 protein. Although MMP7KO mice showed similar numerical increases they were not significant compared to MMP7KO controls. No differences were observed between control WT and MMP7KO mice. Representative western blots for female mice are displayed for NGAL (F) and KIM-1 (I) relative to β-actin. N = 3–10. [Statistical analysis: Kruskal-Wallis test with a Dunn’s multiple comparisons test]. Each point represents an individual animal. Horizontal line indicates combined median. Control = 5% dextrose + room air at 21% O2. NGAL = neutrophil gelatinase-associated lipocalin, KIM-1 = kidney injury marker-1, IL-6 = interleukin-6, IL-1β = interleukin-1β, CXCL-1 = C-X-C motif chemokine ligand 1. (TIF) [file pone.0321349.s012.tif]

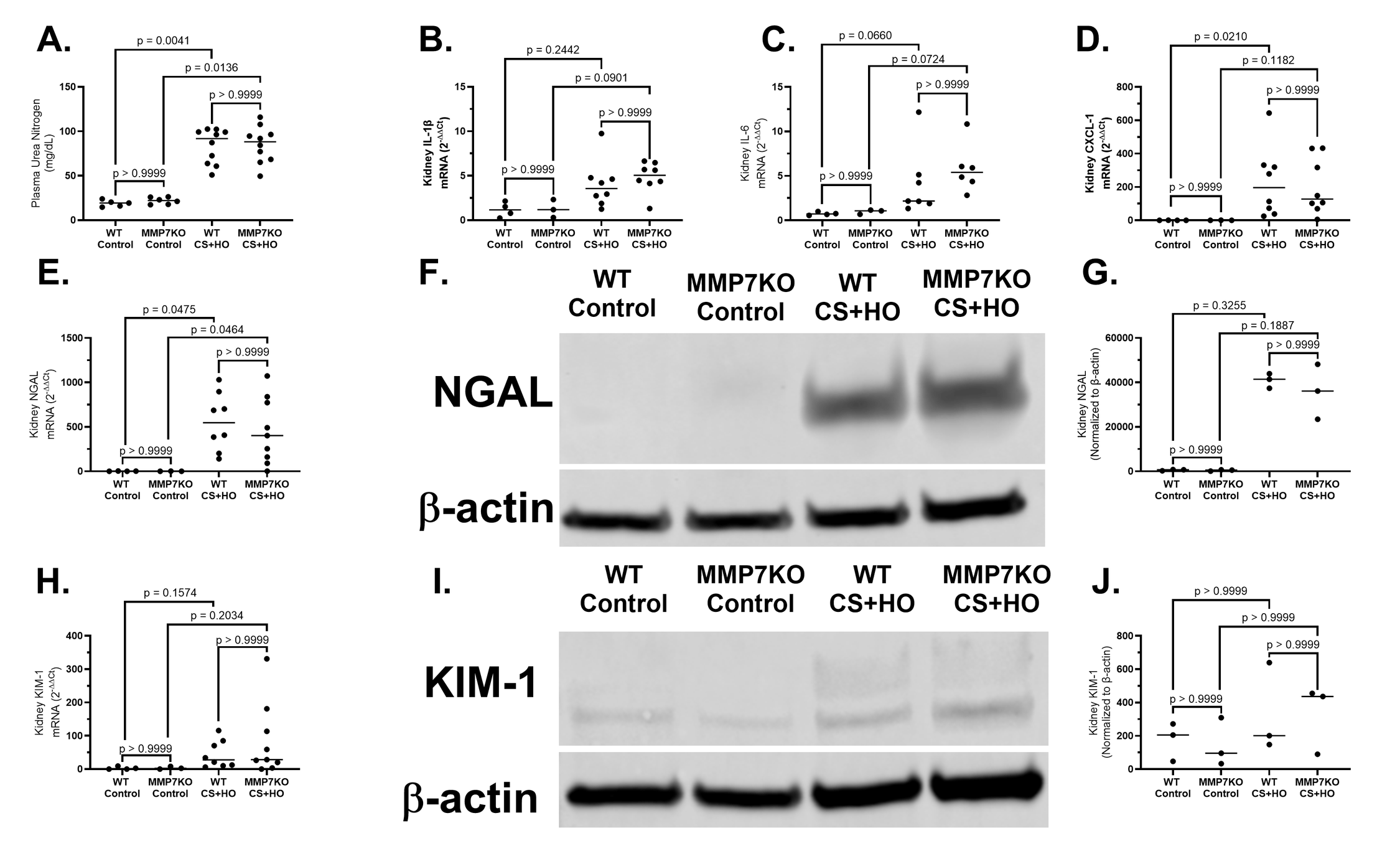

Supplement: S13 Fig — No differences were observed between septic MMP7KO and WT mice for plasma urea nitrogen concentration (A), kidney tissue mRNA levels of IL-1β (B), IL-6 (C), CXCL-1 (D), NGAL (E), or KIM-1 (H), protein levels of NGAL (G) or KIM-1 (J). Septic WT mice showed significantly higher plasma urea nitrogen concentrations, kidney tissue mRNA expression of IL-1β, CXCL-1, and NGAL. Septic WT mice also showed numerically higher kidney tissue mRNA expression of IL-6 and KIM-1 protein. Although MMP7KO mice showed similar numerical increases they were not significant compared to MMP7KO controls. No differences were observed between control WT and MMP7KO mice. Representative western blots for male mice are displayed for NGAL (F) and KIM-1 (I) relative to β-actin. N = 3–10. [Statistical analysis: Kruskal-Wallis test with a Dunn’s multiple comparisons test]. Each point represents an individual. Horizontal line indicates median. Control = 5% dextrose + room air at 21% O2. NGAL = neutrophil gelatinase-associated lipocalin, KIM-1 = kidney injury marker-1, IL-6 = interleukin-6, IL-1β = interleukin-1β, CXCL-1 = C-X-C motif chemokine ligand 1. (TIF) [file pone.0321349.s013.tif]

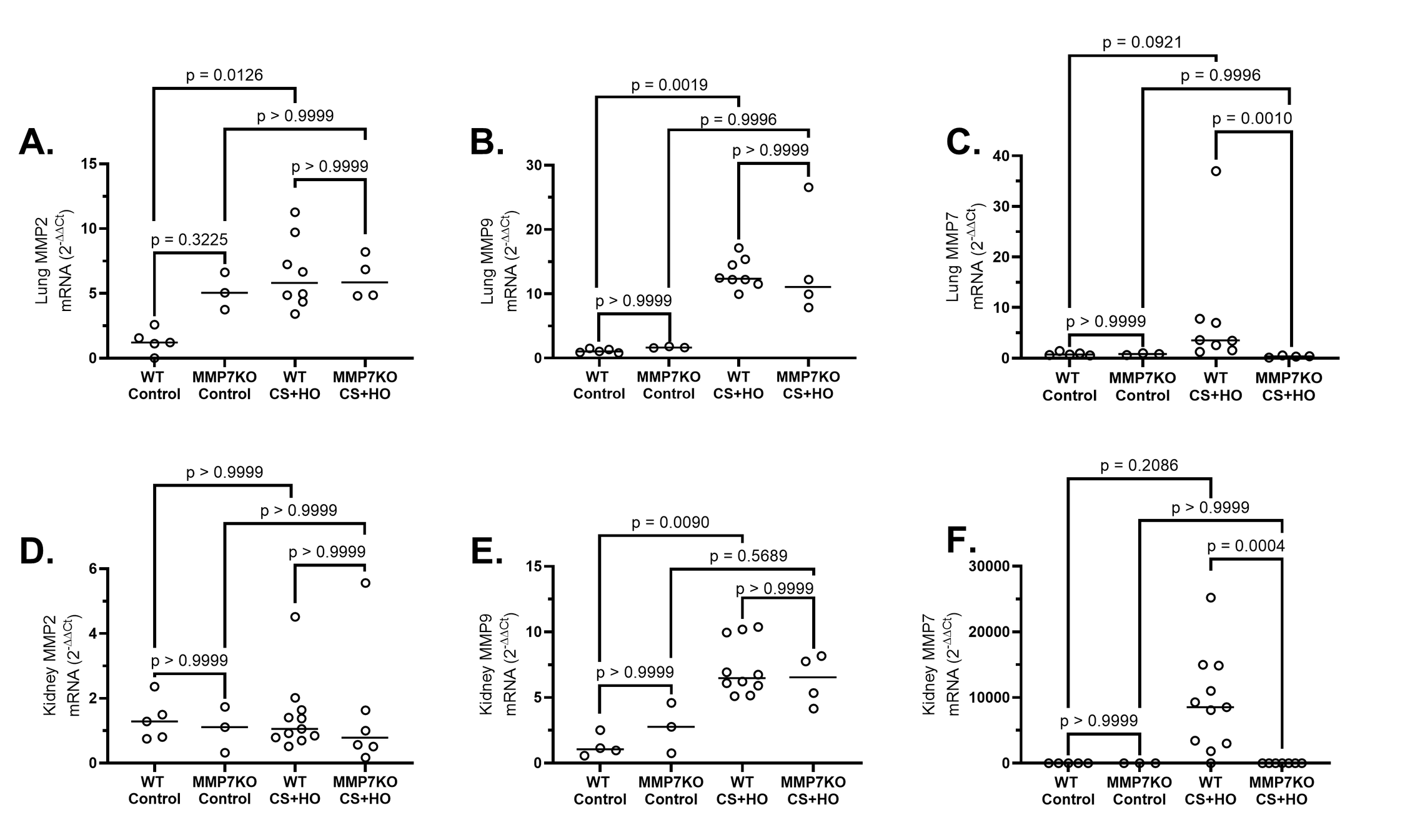

Supplement: S14 Fig — WT Septic mice had significantly higher MMP2 (A) and MMP9 (B), as well as numerically higher MMP7 (C) mRNA expression in lung tissue. WT septic mice also had significantly higher MMP9 (E) mRNA as well as numerically higher MMP7 (p=0.2086; F) in kidney tissue. Septic mice (WT or MMP7KO) did not differ from control mice with respect to kidney mRNA expression of MMP2 (D). Compared to control mice female septic MMP7KO mice did not have significant differences across any of the MMPs measured compared to control MMP7KO mice. Female MMP7KO septic mice had significantly lower lung and kidney MMP7 mRNA expression compared to septic WT mice, but did not differ with regards to MMP9 or MMP2 in either organ. Although no differences were observed between genotypes for control mice, control MMP7KO mice had numerically higher lung MMP2 and kidney MMP9 compared to control WT mice. N = 3–13. [Statistical analysis: Kruskal-Wallis test with a Dunn’s multiple comparisons test]. Each point represents an individual animal. Horizontal line indicates median. Control = 5% dextrose + room air at 21% O2. MMP7 = matrix metalloproteinase-7, MMP9 = matrix metalloproteinase-9, MMP2 = matrix metalloproteinase-2. (TIF) [file pone.0321349.s014.tif]

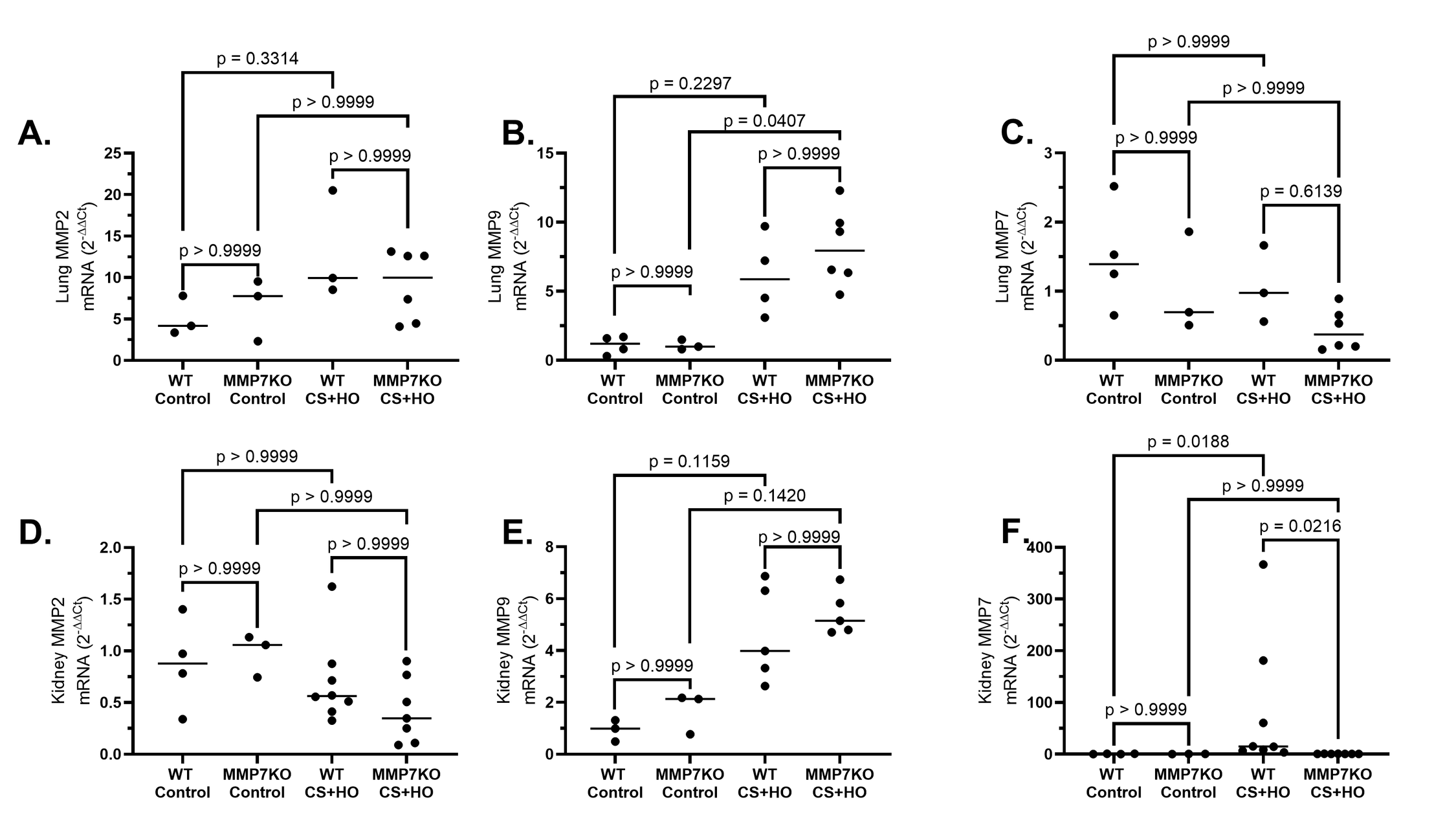

Supplement: S15 Fig — No differences were observed for lung tissue mRNA expression of MMP2 (A), MMP9 (B), or MMP7 (C) between male septic WT and MMP7KO mice. Although septic MMP7KO mice had significantly lower kidney mRNA expression of MMP7 (F) compared to septic WT mice, there were no differences across kidney mRNA expression of MMP2 (D) or MMP9 (E). Septic MMP7KO mice also had significantly higher lung mRNA expression of MMP9 compared to control MMP7KO mice, while WT had numerically higher MMP9 compared to WT controls. Septic mice (both WT and MMP7KO) showed numerical increases in kidney MMP9 mRNA expression compared to control mice. No differences were observed between septic mice and control for MMP2 mRNA expression in the lung or kidney. N = 3–8. [Statistical analysis: Kruskal-Wallis test with a Dunn’s multiple comparisons test]. Each point represents an individual animal. Horizontal line indicates median. Control = 5% dextrose + room air at 21% O2. MMP7 = matrix metalloproteinase-7, MMP9 = matrix metalloproteinase-9, MMP2 = matrix metalloproteinase-2. (TIF) [file pone.0321349.s015.tif]
