# Supplementary material for: Matrix metalloproteinase-7 is dispensable in a mouse model of sepsis-induced acute lung injury
Source: PLoS One. 2025 May 8;20(5):e0321349. doi: 10.1371/journal.pone.0321349 (PMC12061409; doi:10.1371/journal.pone.0321349)
Supplement: S1 Raw Images — (PDF) [file pone.0321349.s017.pdf]

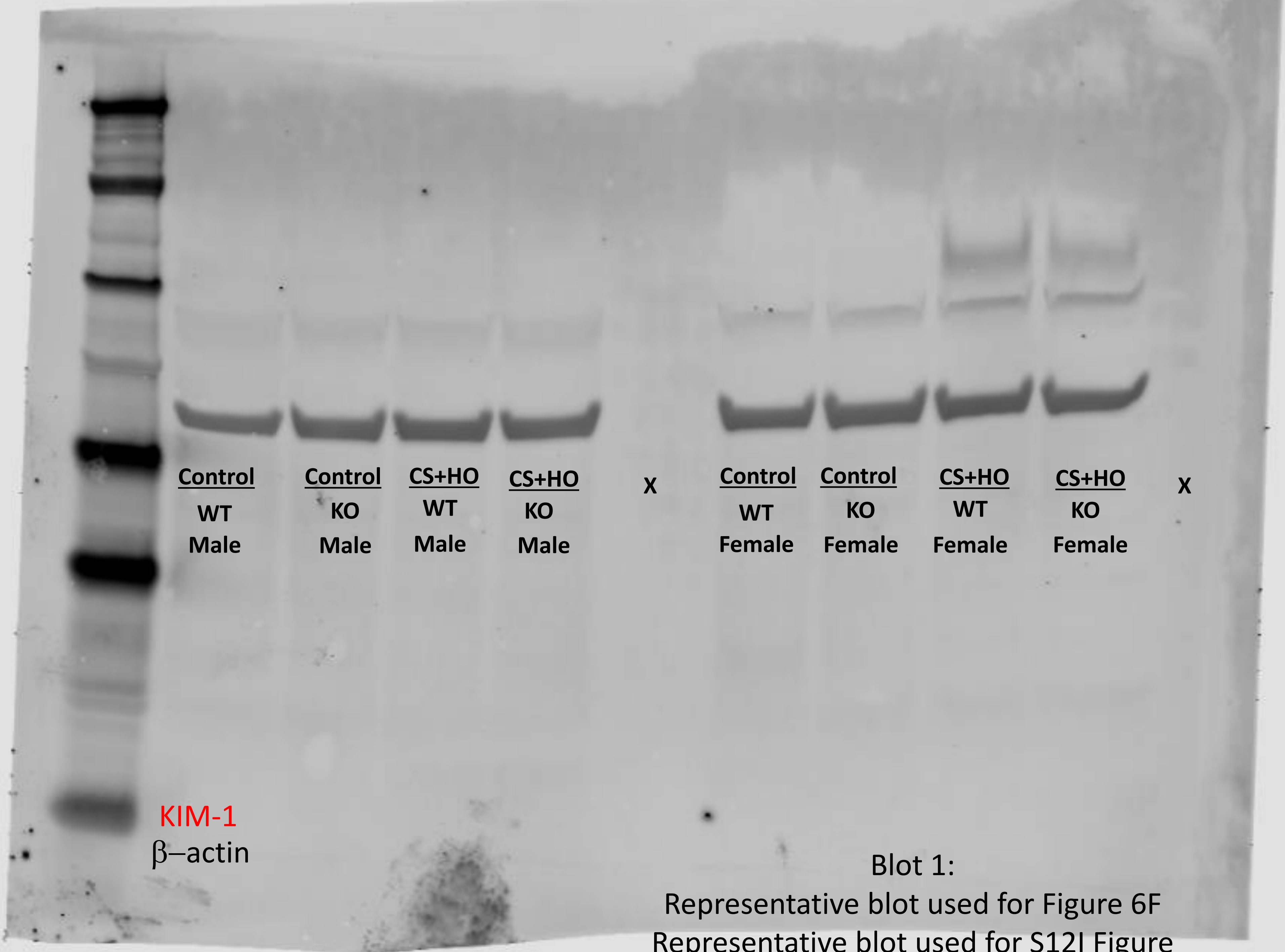

Blot 1:
Representative blot used for Figure 6F
Representative blot used for S12I Figure

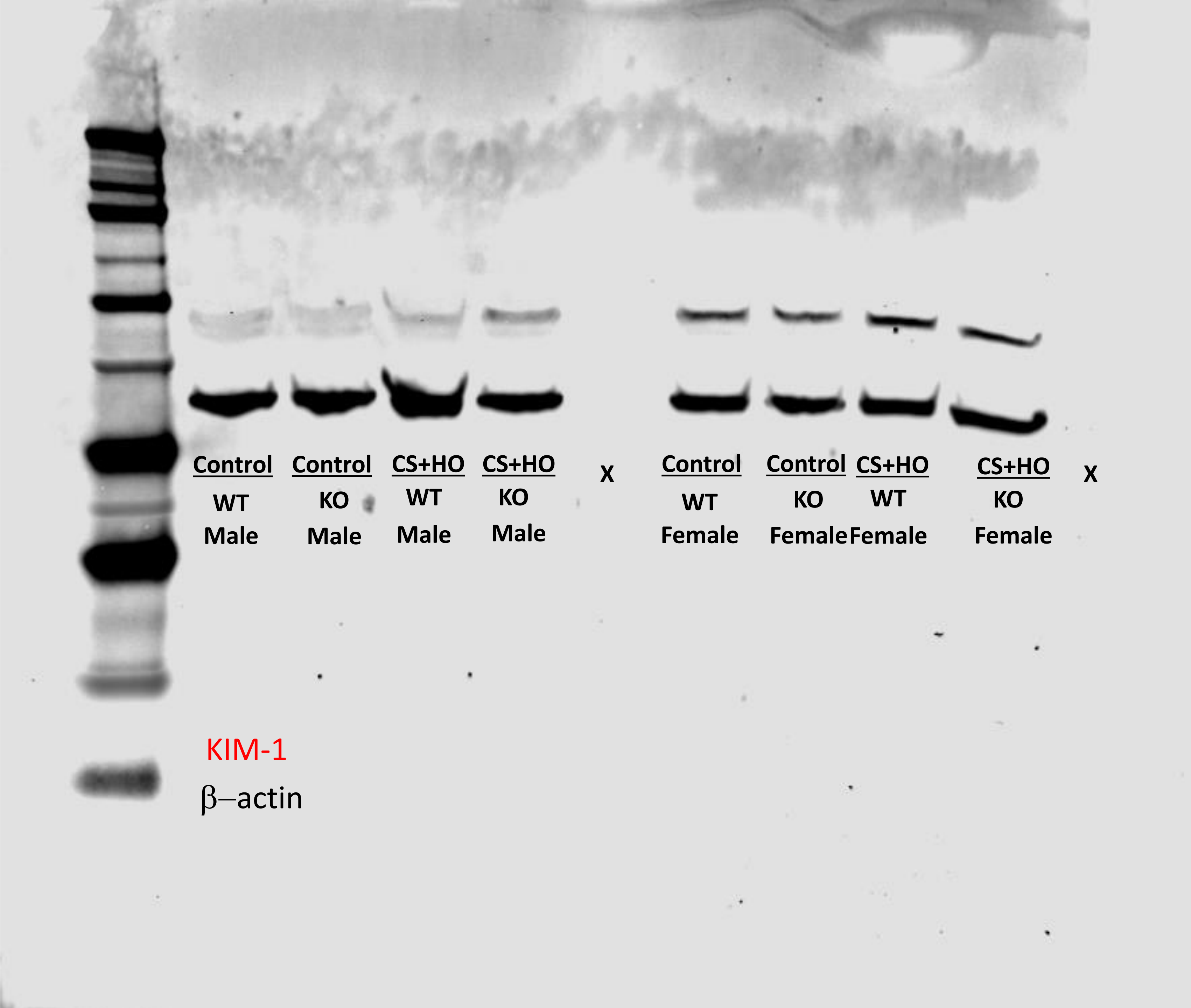
Control WT Male
Control KO Male
CS+HO WT Male
CS+HO KO Male
X
Control WT Female
Control KO Female
CS+HO WT Female
CS+HO KO Female
X
KIM-1
β–actin

Blot 2

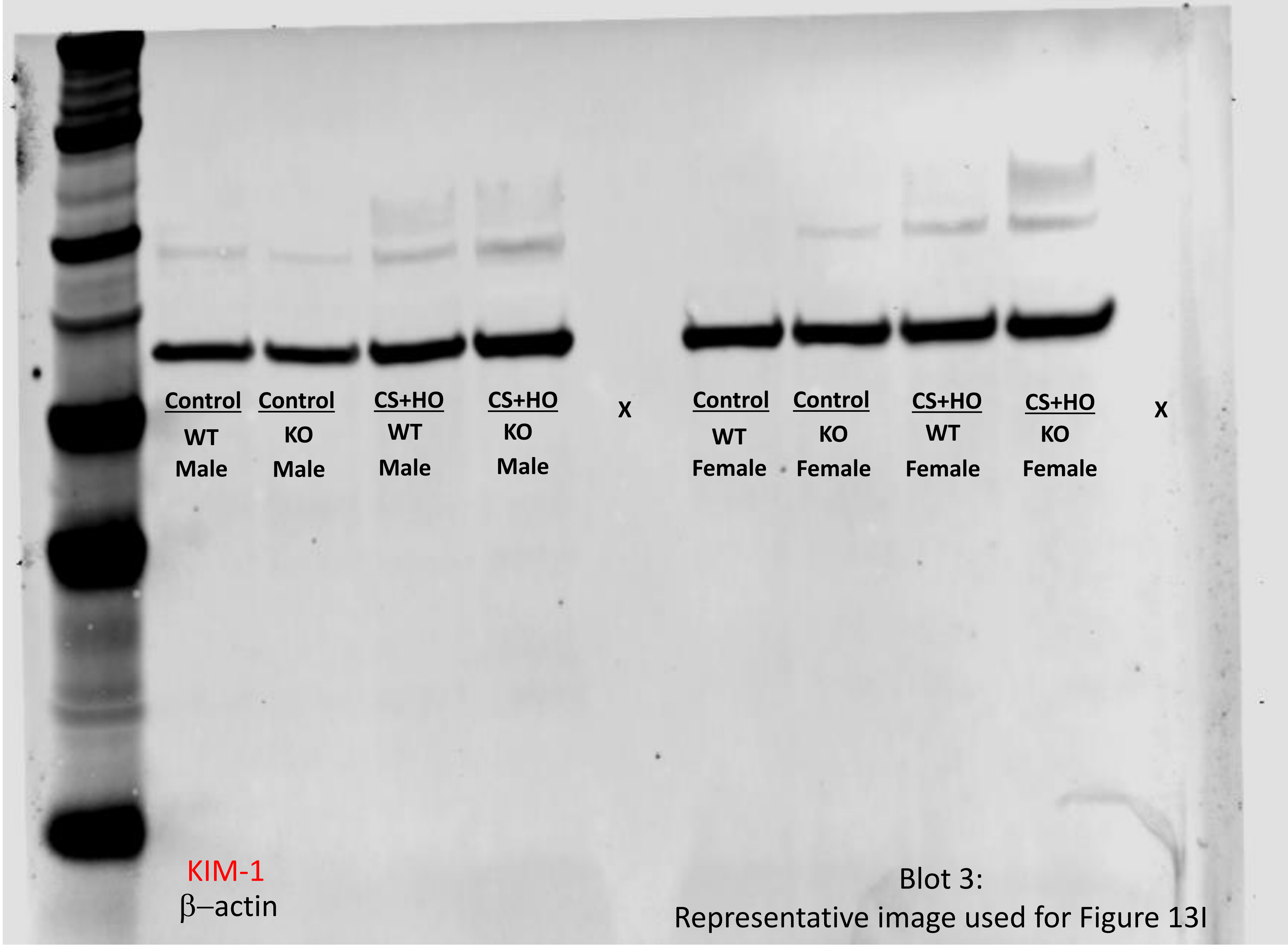

Blot 3:
Representative image used for Figure 13I

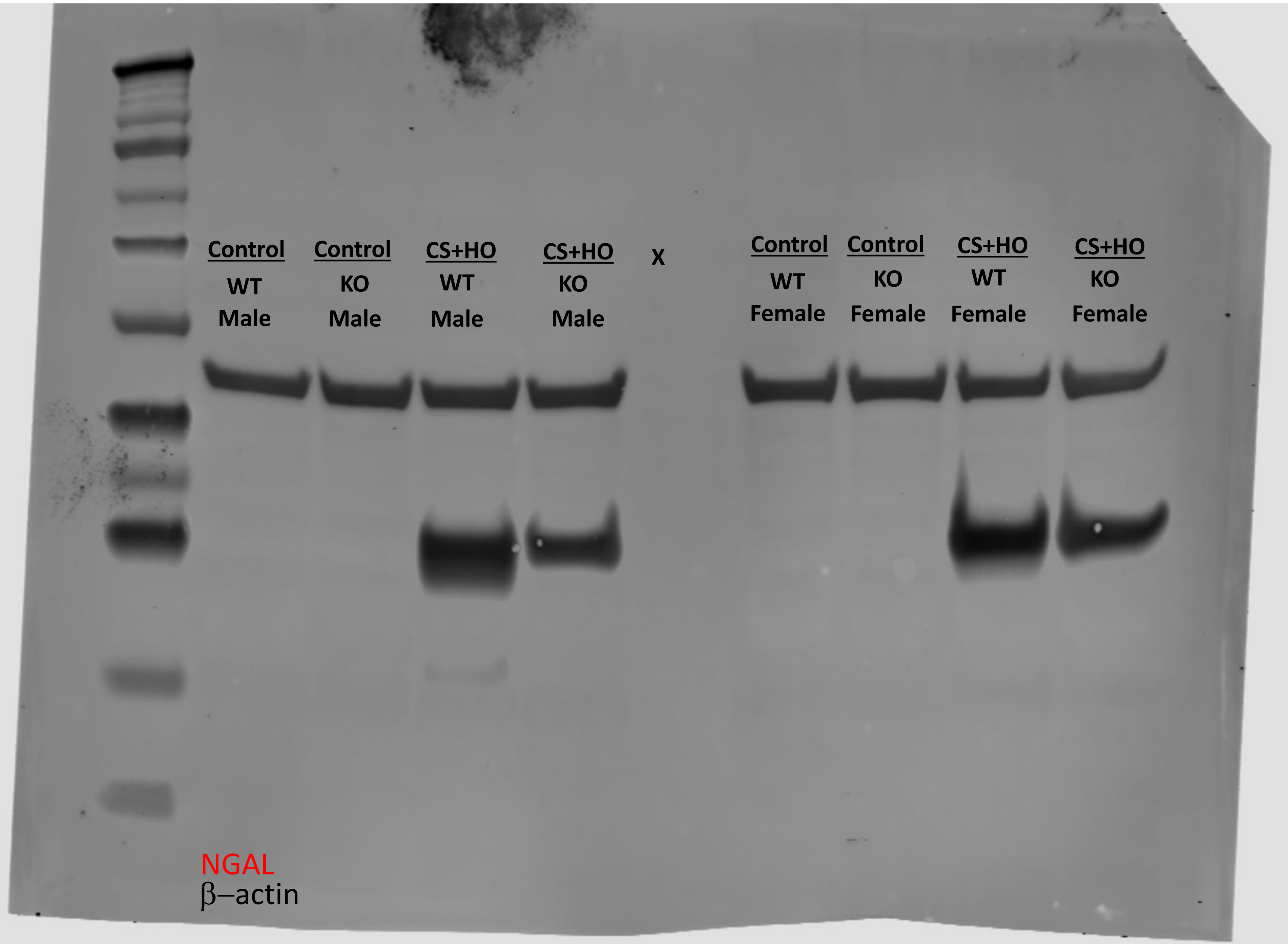
Control WT Male
Control KO Male
CS+HO WT Male
CS+HO KO Male
X
Control WT Female
Control KO Female
CS+HO WT Female
CS+HO KO Female
NGAL
β–actin

Blot 4

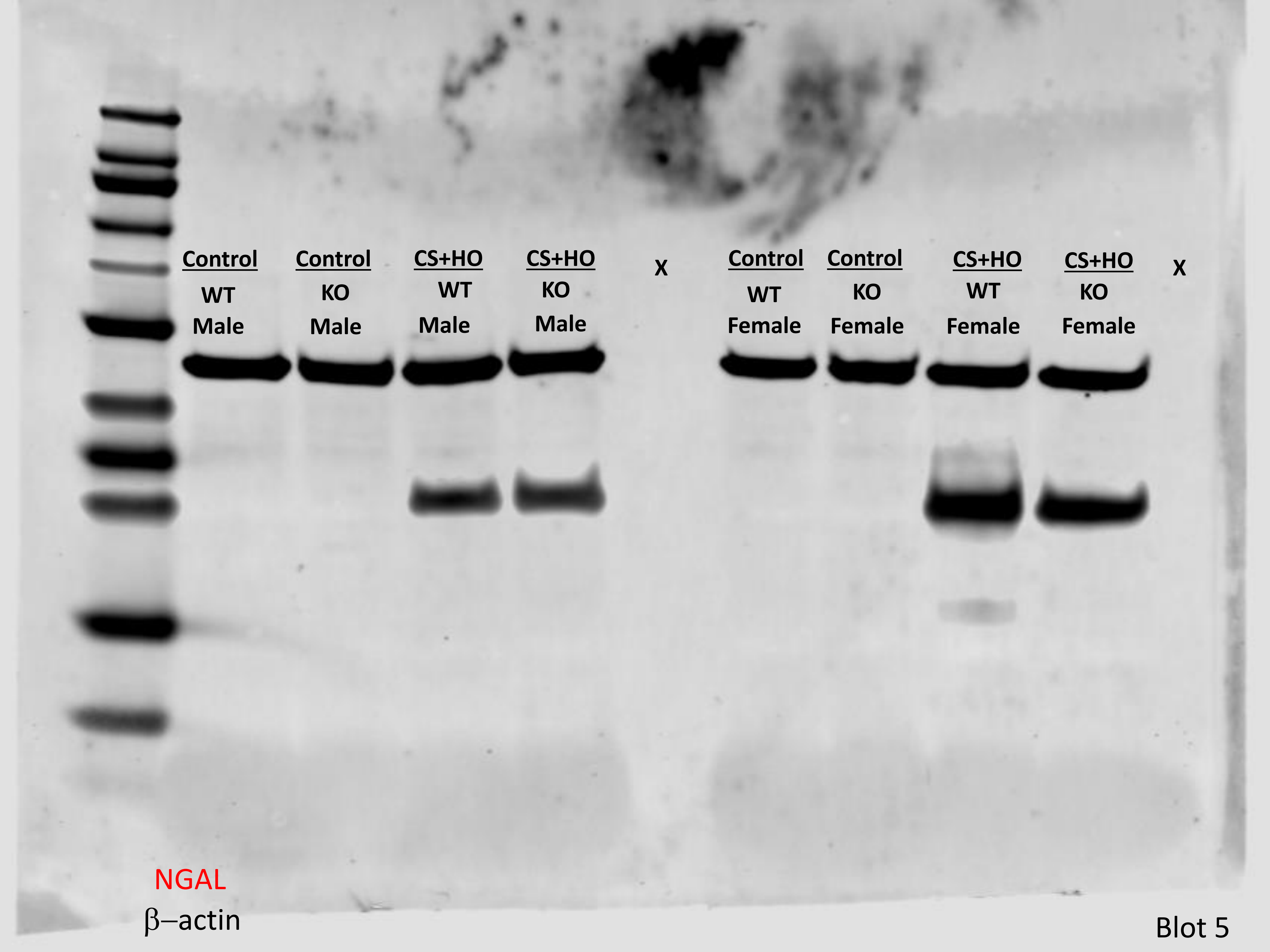
Control
WT
Male
Control
KO
Male
CS+HO
WT
Male
CS+HO
KO
Male
X
Control
WT
Female
Control
KO
Female
CS+HO
WT
Female
CS+HO
KO
Female
X
NGAL
β–actin
Blot 5

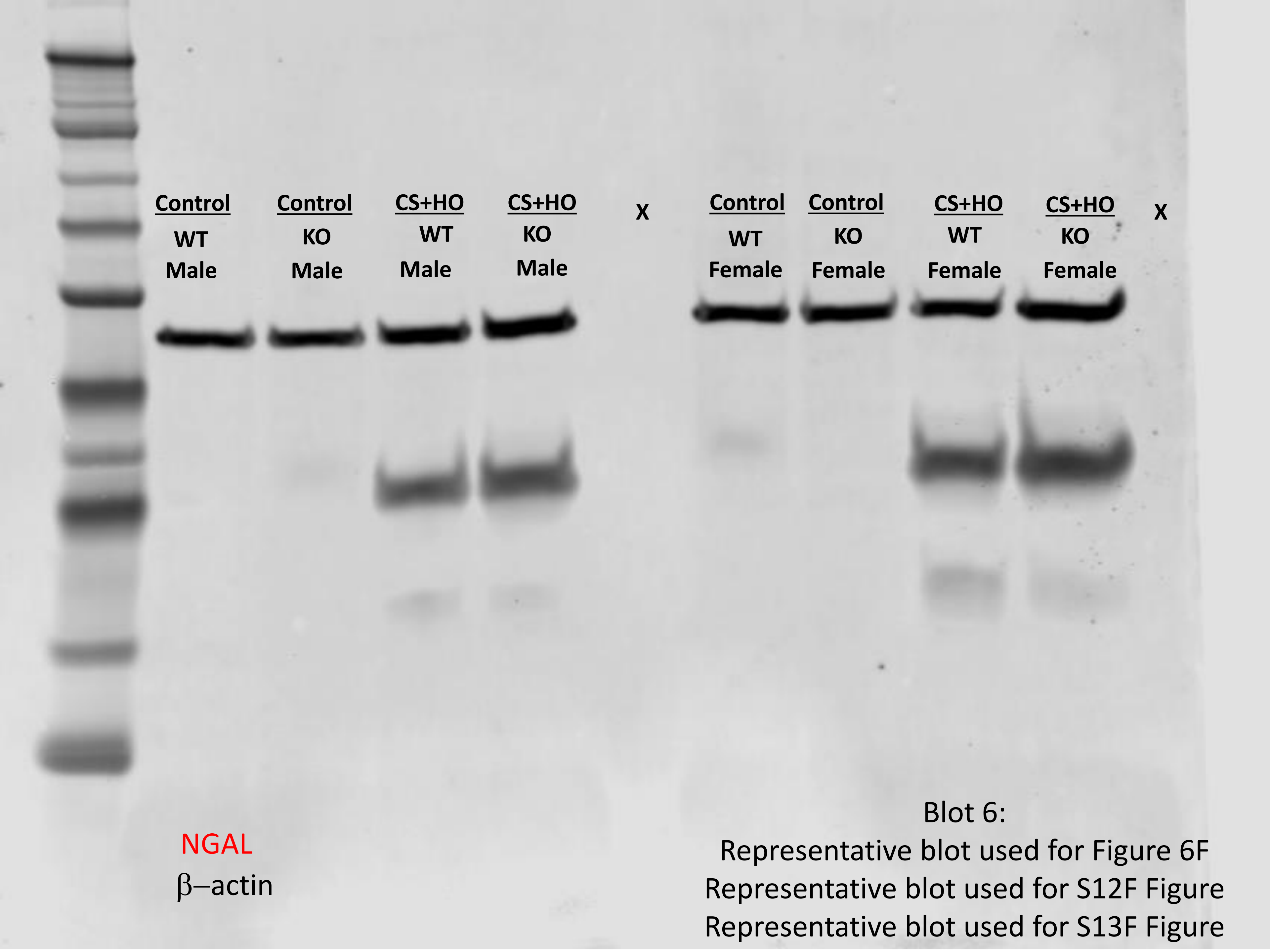

Blot 6:
Representative blot used for Figure 6F
Representative blot used for S12F Figure
Representative blot used for S13F Figure
